# Comparative Structure-Based Virtual Screening Utilizing Optimized AlphaFold Model Identifies Selective HDAC11 Inhibitor

**DOI:** 10.3390/ijms25021358

**Published:** 2024-01-22

**Authors:** Fady Baselious, Sebastian Hilscher, Dina Robaa, Cyril Barinka, Mike Schutkowski, Wolfgang Sippl

**Affiliations:** 1 Department of Medicinal Chemistry, Institute of Pharmacy, Martin-Luther-University of Halle-Wittenberg, 06120 Halle (Saale), Germany; f_noshy@yahoo.com (F.B.); sebastian.hilscher@pharmazie.uni-halle.de (S.H.); dina.robaa@pharmazie.uni-halle.de (D.R.); 2 Institute of Biotechnology of the Czech Academy of Sciences, BIOCEV, 252 50 Vestec, Czech Republic; cyril.barinka@ibt.cas.cz; 3 Charles Tanford Protein Center, Department of Enzymology, Institute of Biochemistry and Biotechnology, Martin-Luther-University of Halle-Wittenberg, 06120 Halle (Saale), Germany; mike.schutkowski@biochemtech.uni-halle.de

**Keywords:** AlphaFold, HDAC11, virtual screening, modelling, in vitro assay, pharmacophore, docking, molecular dynamics simulation

## Abstract

HDAC11 is a class IV histone deacylase with no crystal structure reported so far. The catalytic domain of HDAC11 shares low sequence identity with other HDAC isoforms, which makes conventional homology modeling less reliable. AlphaFold is a machine learning approach that can predict the 3D structure of proteins with high accuracy even in absence of similar structures. However, the fact that AlphaFold models are predicted in the absence of small molecules and ions/cofactors complicates their utilization for drug design. Previously, we optimized an HDAC11 AlphaFold model by adding the catalytic zinc ion and minimization in the presence of reported HDAC11 inhibitors. In the current study, we implement a comparative structure-based virtual screening approach utilizing the previously optimized HDAC11 AlphaFold model to identify novel and selective HDAC11 inhibitors. The stepwise virtual screening approach was successful in identifying a hit that was subsequently tested using an in vitro enzymatic assay. The hit compound showed an IC_50_ value of 3.5 µM for HDAC11 and could selectively inhibit HDAC11 over other HDAC subtypes at 10 µM concentration. In addition, we carried out molecular dynamics simulations to further confirm the binding hypothesis obtained by the docking study. These results reinforce the previously presented AlphaFold optimization approach and confirm the applicability of AlphaFold models in the search for novel inhibitors for drug discovery.

## 1. Introduction

Histone deacetylases (HDACs) form a protein family responsible for catalyzing the elimination of acetyl groups from lysine residue of histone proteins as well as other substrates [1]. The histone deacetylase family is classified into four main classes, three of which are constituted by eleven zinc-dependent HDACs, namely, class I (HDAC1, 2, 3 and 8), class IIa (HDAC4, 5, 7 and 9), class IIb (HDAC6 and 10), and class IV (HDAC11) [2].

HDAC11, the sole member of class IV of the HDAC family, is the smallest member of the family and one of the least studied HDAC subtypes [3,4]. It is expressed in multiple organs, including the heart, kidney, brain tissues, skeletal muscles, and gall bladder [4,5]. Evidence has demonstrated that HDAC11 is involved in various physiological processes such as modulation of the immune system [6,7] and maintaining genomic integrity [8]. It was also evident that HDAC11 is connected to some pathological processes and represents a potential target for the treatment of several diseases, including multiple sclerosis, viral infections, and obesity-related diseases [9,10,11]. HDAC11 was also found to be involved in the modulation of cancer growth and is overexpressed in different cancer forms [12,13,14,15,16,17,18,19]. For example, inhibition of HDAC11 showed beneficial effects in neuroblastoma cells [20], suggesting that HDAC11 represents a promising target for the treatment of some cancer forms.

HDAC11 has been found to have robust fatty acid deacylase activity. This activity is more than 10,000-fold more efficient than the deacetylase activity, suggesting that this activity may be the major activity of the enzyme in vivo [21,22,23].

To date, only a few selective HDAC11 inhibitors have been reported. Hydroxamic acid-based inhibitors include FT895 [24], the only weakly active MIR002 [25], and the recently developed inhibitor BP94 [26]. FT895 showed beneficial effects in reducing non-small cell lung cancer cells’ viability [27], while BP94 was able to ameliorate neuropathic pain in mouse model [26]. Due to its preference to remove long-chain fatty acyl groups, it has been postulated that HDAC11 contains a hydrophobic pocket near its catalytic Zn^2+^ center. Therefore, inhibitors containing long alkyl chains have been described, such as SIS17 [28], which contains an alkyl hydrazide moiety and inhibits HDAC11 in vitro in the submicromolar range. Alkyl hydrazides have also recently been described for other HDACs, such as HDAC3 and HDAC8, as novel zinc binding groups. [29,30]. Similarly, the trapoxin A analog TD034 [31] possesses a long alkyl chain that might be the reason for the observed HDAC11 selectivity [31].

No crystal structure of HDAC11 has been reported, and its catalytic domain shows low sequence identity (<30%) when compared to the primary sequences of the catalytic domains available in the PDB databank for other human HDAC isoforms. This fact complicates the conventional template-based homology modeling [32].

AlphaFold is a machine learning approach for predicting the 3D structures of proteins with atomic accuracy even in absence of known similar structures [33]. A database containing the 3D structures of the whole human proteome was built by AlphaFold [34]. The models from AlphaFold should be carefully considered when used for structure-based drug design studies because the folding is predicted in absence of small molecules like water molecules, ligands, and cofactors.

In a recent study by Ren et al. [35], AI-driven molecular generation was combined with utilization of the AlphaFold model for the aim of drug discovery for cyclin-dependent kinase 20 (CDK20). In this study, modification of the AlphaFold model by removing the C-terminus which was blocking the solvent-exposed region of the protein and occupying the ATP binding pocket through Arg305 was performed in order to make the model usable. In another study, Zhu et al. [36] utilized a similar approach to successfully design new inhibitors for salt-inducible kinase 2 (SIK2).

The two studies discussed above used AlphaFold models for protein targets sharing reliable sequence identity with other proteins within the same family for which crystal structures are available and utilized AI-driven molecular generation techniques rather than docking. Several other studies addressed the usability of AlphaFold models for docking [37,38,39,40,41] and real-world virtual screening scenarios [39,42,43]. One of these studies assessed the usability of AlphaFold structures predicted while excluding structural templates with more than 30% identity, thus imitating a virtual screening process with a model based on low prior structural information. Results from these studies demonstrated a worse performance of the AlphaFold models compared to crystal structures, suggesting that using unmodified AlphaFold models is not an ideal scenario. This poorer performance could be due to incorrect binding site geometry, resulting from minor variation at the side-chain level or larger variation of the backbone, suggesting that post-modeling or optimization is required to obtain more realistic holo models [38,39,40,41,42,43].

In agreement with these results, it was demonstrated that optimization of the binding site by inducing flexibility or manually modifying of the low confidence regions could enhance the docking results [37,39,40,44]. In our recent work, we showed that binding site optimization of the HDAC11 AlphaFold model by adding the catalytic zinc ion and performing minimization in the presence of inserted ligands resulted in a model that could be used for docking of the known selective HDAC11 inhibitors FT895, MIR002, and SIS17 [32].

In the current study, we present an application of an optimized AlphaFold model for virtual screening while addressing HDAC subtype selectivity [45]. We demonstrate herein that our previously optimized HDAC11 AlphaFold model was successfully utilized for picking a selective hit through a comparative virtual screening approach. In the developed multistep screening, various approaches, including structure-based pharmacophore screening as pre-filtering of large databases, ligand docking, pose filtering, and prioritization, were applied as described in Section 3. To experimentally confirm the virtual screening results, the most promising hit was synthesized and tested in vitro using different HDAC subtypes. In addition, we analyzed the predicted binding mode from docking by means of molecular dynamics (MDs) and MetaDynamics simulations.

## 2. Results and Discussion

### 2.1. Dataset Selection and Curation

**Figure 1:**
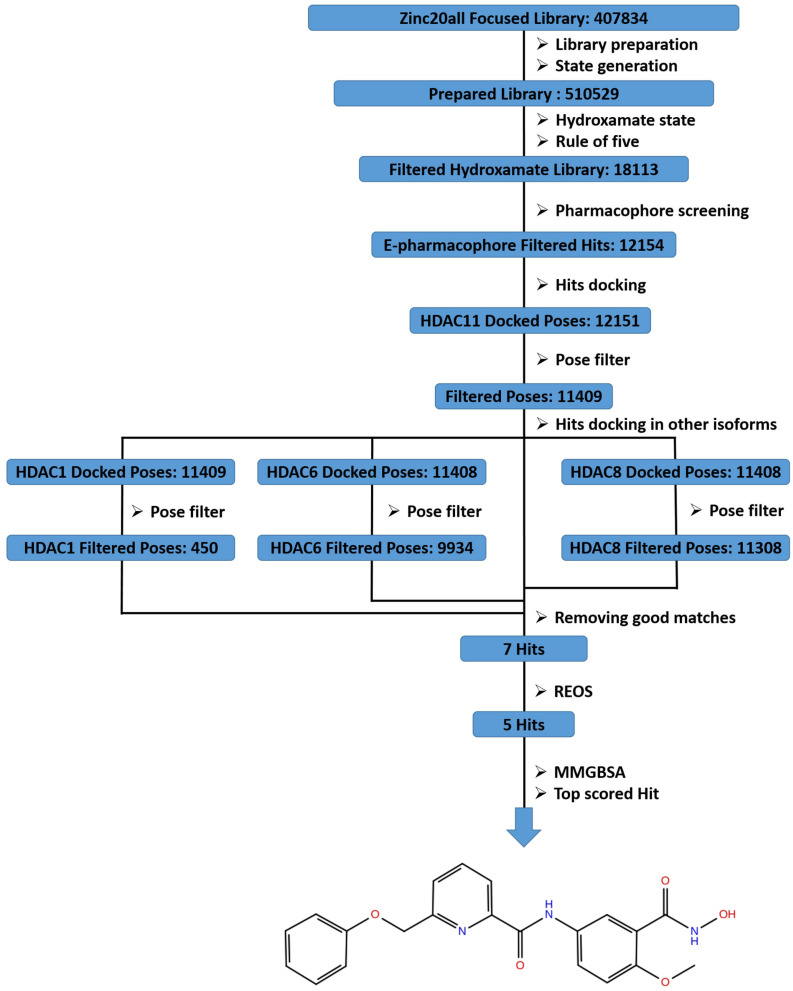
Workflow of the stepwise virtual screening.

Hydroxamates comprise a well-defined and characterized pharmacophore for HDAC inhibitors and are considered the most commonly used zinc binding group in HDAC inhibitors [46,47]. Some of the inhibitors bearing the hydroxamate scaffold, such as vorinostat (SAHA), belinostat (PXD-101), and panobinostat (LBH589), have been approved by the FDA in the past for the treatment of hematological malignancies [48]. Benzohydroxamates constitute an important class of HDAC inhibitors, and their development entails an active field within inhibitor design for several HDAC subtypes [47]. ZINC20 is a publicly available database that includes nearly two billion compounds, in 2D and 3D downloadable formats, through a website that allows for rapid analogue searching [49]. Initially, a focused database of 407,834 benzohydroxamates was acquired from the ZINC20 database. The library was further prepared by generating possible ionization states at physiological pH 7.0 ± 2.0. The preparation step resulted in a library that contained 510,529 ligands with various ionization states; this library was then subjected to filtration to select the ligands with a hydroxamate state only. The Lipinski rule of five is an important early measure for identifying bioavailable drug-like candidates. According to this rule, a compound must possess the following properties: molecular weight < 500 Da, logP < 5, H-bond donors < 5, and H-bond acceptors < 10. To further select drug-like molecules, the prepared library was filtered to remove any molecule that violated Lipinski’s rule of five [50,51]. The initial curation resulted in a library of 18,113 ligands. The multistep virtual screening process was then performed as presented in the workflow (Figure 1).

### 2.2. Virtual Screening

**Figure 2:**
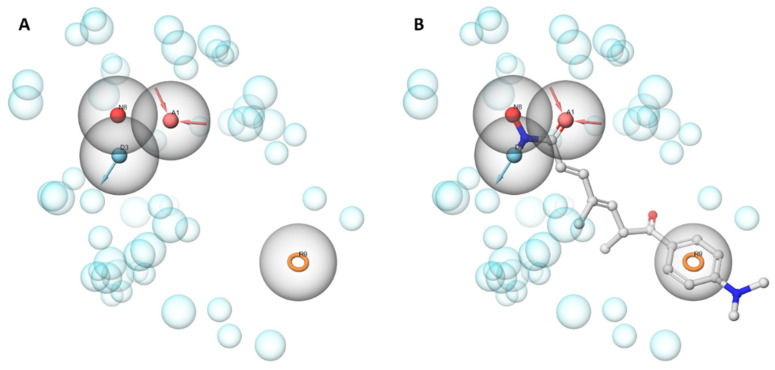
E-pharmacophore model. (**A**) Pharmacophore features: HB donor represented as cyan arrows, HB acceptor as orange arrows, negative features as red dots, and aromatic features as orange rings. Allowed volumes for pharmacophore features are shown as grey spheres. Excluded volumes are represented as cyan transparent spheres and feature matching tolerance as grey transparent spheres. (**B**) Superposition of the inhibitor TSA on the features of the generated hypothesis. Ligand is represented as stick model (grey = carbon, blue = nitrogen, red = oxygen).

The E-pharmacophore module implemented in Schrödinger’s PHASE automatically generates a pharmacophore hypothesis that is based on the complementarity of the protein and ligand features from a protein–ligand complex. This involves using Glide XP scoring terms to determine which features contribute the most to the binding. The hypothesis obtained from using the previously optimized complex of TSA and the HDAC11 AlphaFold model exhibited four features (Figure 2), namely, a hydrogen bond acceptor feature assigned for the carbonyl-O, a hydrogen bond donor assigned to the NH, a negative feature for the deprotonated hydroxyl group of the hydroxamate zinc binding group, and an aromatic feature for the phenyl capping group. Excluded volumes that are based on the occupation of space by protein atoms were also added. Pharmacophore screening was performed to select the ligands that matched the four features, with the aim of filtering out very small ligands/fragments as well as compounds larger than what could be accommodated in the HDAC11 pocket. Thus, the pharmacophore formed by the excluded volumes was primarily used to reduce the very large number of compounds for the subsequent and more computationally demanding docking method.

**Figure 3:**
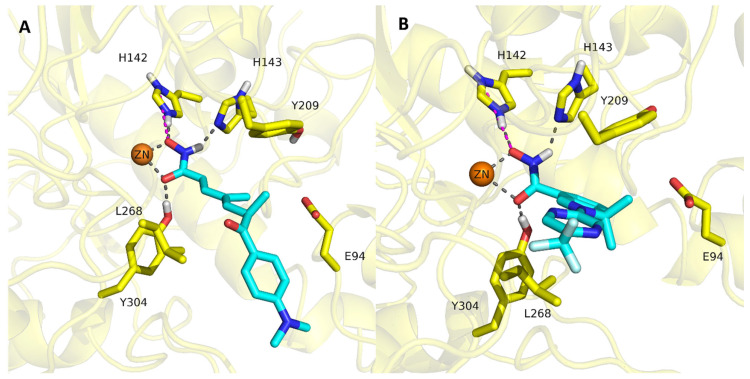
(**A**) Minimized pose of TSA in HDAC11-optimized AlphaFold model. (**B**) Docked pose of FT895 in the optimized HDAC11 AlphaFold model. The protein backbone is represented as yellow cartoon, the interacting binding site residues as yellow sticks, zinc ion as orange sphere, and the ligands as sticks (cyan = carbon, blue = nitrogen, red = oxygen, grey = polar hydrogens). Hydrogen bonds and coordination bonds are represented as grey dashed lines and ionic interactions as magenta dashed lines.

The pharmacophore screening step was effective and could filter out 5959 compounds. Docking-based virtual screening of the remaining 12,154 structures was then performed using the grid generated from the HDAC11-TSA-optimized AlphaFold model. In our previous study, we successfully obtained four optimized complexes by minimization of the HDAC11 AlphaFold model with previously reported active ligands of HDAC11, for which X-ray crystal structures with HDAC8 are available in the protein databank (PDB). The selection of the TSA-HDAC11 complex for the virtual screening was based on the results obtained from the previous study, since it showed the best performance regarding the docking of the selective inhibitor FT895 (Figure 3) and was further utilized in docking of other selective inhibitors such as MIR002 and SIS17. Almost all of the hits from the pharmacophore screening step were able to pass the docking-based screening. Furthermore, filtration of the obtained docking poses was performed to select the ligands that can show a bidentate chelation mode to the catalytic zinc ion. Pose filtration was performed utilizing the distances between the chelator carbonyl and hydroxyl oxygen atoms of the hydroxamate moiety to the zinc ion. Compounds showing distances more than a cut off of 2.6 Å between any of the chelator atoms and the zinc ion were removed.

With the aim of searching for selective HDAC11 ligands, a comparative docking-based virtual screening approach was then applied. The hits obtained from the docking in HDAC11 which could pass the pose filtration step were then screened by docking into HDAC1, HDAC6, and HDAC8 crystal structures. The obtained hits from every screening were further subjected to pose filter screening. Ligands which could show correct docking pose with bidentate chelation of the catalytic zinc ion in any of HDAC1, HDAC6, and HDAC8 were removed from the HDAC11 hit list. For HDAC6, ligands which could chelate the zinc ion in a monodentate fashion were also removed. This step was very effective and could filter out most of the compounds, leaving only seven compounds (Table S1, Supplementary Materials) that could show a correct chelation mode in HDAC11 but not in any of the other isoforms.

Rapid elimination of swill (REOS) [52,53] filter was then applied to remove compounds containing reactive or toxic moieties which might also interfere with biological assays. Two compounds containing nitro groups were removed by using this filter. Interestingly, the final five hits (Table 1) all bear a methoxy, ethoxy, or chloro substituent on the ortho position of the hydroxamate moiety, which indicates that substitution at this position might represent a selectivity determinant for HDAC11 inhibition.

In the last step of the virtual screening workflow, the five final hits were prioritized through MM-GBSA calculations. MM-GBSA calculations showed that the top-ranked molecule is ZINC000028464438 (**9**), which bears a methoxy group as ortho substitution to the hydroxamate moiety and an amide linker in the meta-position. It is worth noting that a selective HDAC11 inhibitor (PB94) was recently presented by Bai et al. [26]. Based on the structure–activity relationship, the authors reported that a methoxy group in the ortho position of their developed benzohydroxamate inhibitors is a key factor for HDAC11 selectivity, which is in agreement with our results from the virtual screening.

### 2.3. In Vitro Enzymatic Evaluation

**Figure 4:**
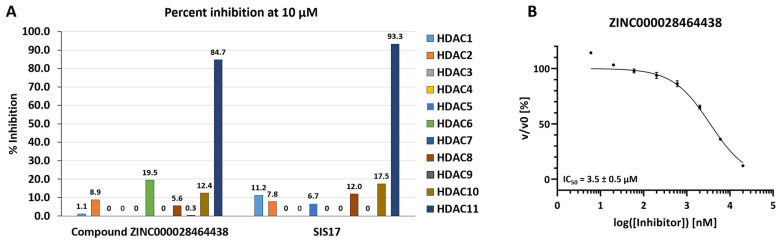
(**A**) Relative inhibition of enzymatic activity for all HDAC subtypes at 10 µM of **9** (ZINC000028464438) and SIS17. (**B**) Determination of IC_50_ value of **9** (ZINC000028464438) for HDAC11.

Due to the unavailability of the top-ranked hit ZINC000028464438, we decided to resynthesize the compound (**9**) as reported [54]. We purified it, confirmed the structure by NMR and MS, and tested it at a concentration of 10 µM against HDAC11 as well as all other HDAC subtypes (HDAC1–10) to determine the selectivity. The synthesis and analytical characterization are described in detail in Section 3. Compound **9** showed inhibition of around 85% in the enzymatic activity for HDAC11, almost no inhibition for nearly all HDAC subtypes, and only around 20% inhibition of HDAC6 (Figure 4A). Interestingly, the findings from the in vitro screening confirm the results obtained from the theoretical study, as the hit compound was not able to adopt reasonable poses in any of HDAC1, HDAC6, and HDAC8. On the other hand, a perfect pose with a bidentate chelation mode that also demonstrates the expected interactions of a benzohydroxamate-based HDAC inhibitor was observed in HDAC11 and proved to be stable during MD simulations. These results further confirm that HDAC11 can accommodate such bulkier substitutions in the ortho position of the benzohydroxamate moiety of the inhibitor, providing a unique feature that can be used to target isoform selectivity when designing new inhibitors.

Furthermore, the IC_50_ for HDAC11 was determined to be about 3.5 µM (Figure 4B). While this virtual screening hit showed only moderate HDAC11 inhibitory activity, it still can be considered a promising hit compound due to the good selectivity. Further chemical optimization is required that might include manipulation of the size and structure of the ortho substituent at the benzohydroxamate moiety, changing the position and structure of the amide linker, or changing the structure and decorations of the capping group. The obtained results can be assessed in the light of capabilities of virtual screening and the role it plays for hit identification and finding new scaffold leads by screening large compound libraries, a process that is commonly followed by lead optimization. We included the well-characterized HDAC11 inhibitor SIS17 as a reference compound in our enzyme inhibition assay, and it showed IC_50_ of 0.17 µM, which is in line with reported data [28] (Figure 4A and Figure S6).

### 2.4. Analysis of the Docked Poses

**Figure 5:**
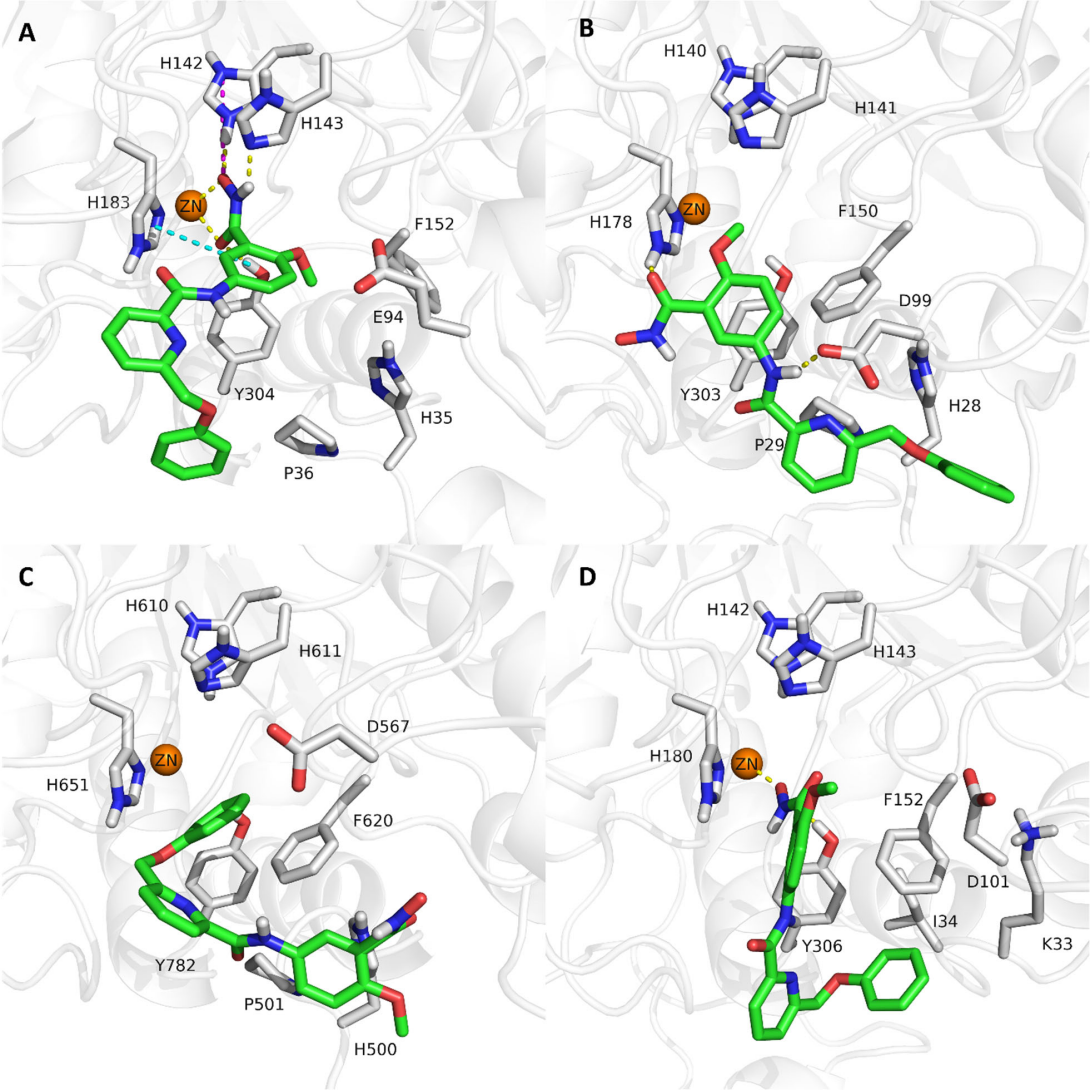
Docked poses of ZINC000028464438 (**9**). (**A**) HDAC11. (**B**) HDAC1. (**C**) HDAC6. (**D**) HDAC8. The protein backbone is shown as white cartoon, zinc ion as orange sphere, the binding site residues as grey sticks, and the ligands as sticks (green = carbon, blue = nitrogen, red = oxygen, grey = polar hydrogens). Coordination and hydrogen bonds are shown as yellow dashed lines, π–π interactions as cyan dashed lines, and the ionic interactions as magenta dashed lines.

Analyzing the docked poses of the confirmed hit revealed that the obtained pose of the hit compound in the optimized HDAC11 AlphaFold model (Figure 5) showed bidentate chelation, with distances of 2.41 Å and 2.17 Å between the zinc ion and the carbonyl and hydroxyl oxygen atoms of the hydroxamate moiety, respectively. A salt bridge to His142 as well as hydrogen bond interactions with His143 and Tyr304 were observed. The ligand also demonstrated π–π interactions between the phenyl ring of the benzohydroxamate and His183. The phenoxymethyl capping group adopts a bent conformation and is directed towards loop1. For HDAC1, the hit ligand showed a pose in which no metal chelation was observed, as the hydroxamate moiety could not reach the zinc ion in the depth of the binding pocket, barely reaching His178, with which the ligand forms a hydrogen bond through the hydroxyl oxygen of the hydroxamate moiety. Another hydrogen bond was observed between the NH of the amide linker and Asp99 side chain. In HDAC6, the docking resulted in a flipped orientation, with the hydroxamate moiety facing the solvent, which indicates that the ligand could not fit into the binding site. No interactions could be observed for the obtained pose in HDAC6. The hit ligand could not show the bidentate zinc chelation commonly observed for co-crystallized HDAC8 inhibitors.

In previous studies, we performed a structural comparison of the optimized HDAC11 AlphaFold model with HDAC6 and HDAC8 as candidates of class I and class II HDACs [32]. The comparison showed that the folding of loop 3 of HDAC11 is more similar to HDAC8, suggesting the formation of the so-called foot pocket in HDAC11, similarly to HDAC8. Thus, the HDAC11 model shows a large foot pocket that justifies the binding of ligands with long alkyl chains, such as the alkyl hydrazide derivative SIS17. The entrance of the foot pocket in HDAC11 is formed by the residues Gly139, Gly140, and Phe141, whereas in HDAC8, the Phe141 is replaced by the bulkier residue Trp141. In HDAC6, loop 3 residues are replaced by the bulkier Pro607 and Pro608 as well as the larger residue Arg606. In addition, the Arg606 side chain is directed towards loop 1, forming polar interactions with Glu50, thus causing loop 3 to fold in the opposite direction and blocking the formation of the foot pocket in HDAC6.

**Figure 6:**
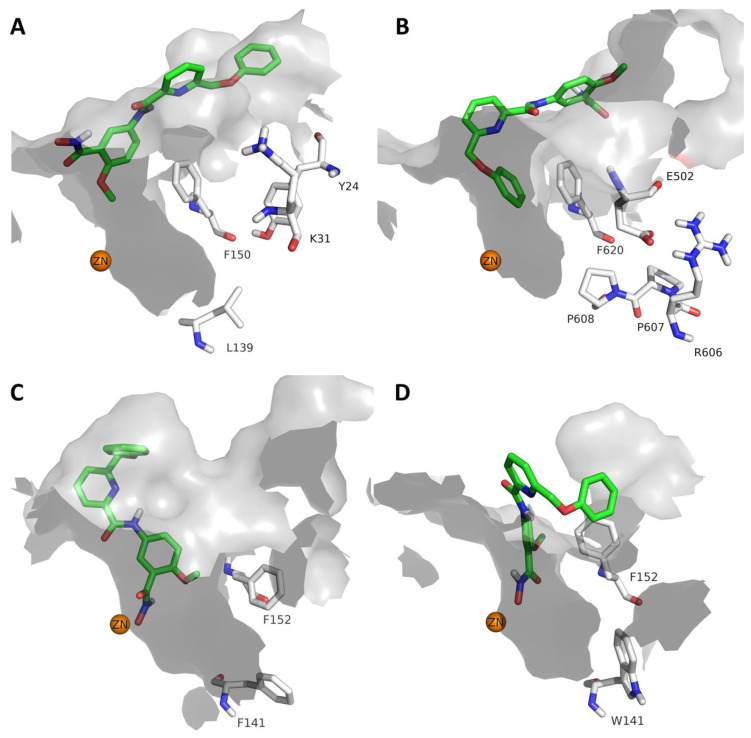
Docking poses of the hit compound **9** and demonstration of the binding site shape and size. (**A**) HDAC1 (PDB ID 5ICN), (**B**) HDAC6 (PDB ID 5EDU), (**C**) HDAC11 (AlphaFold model), (**D**) HDAC8 (PDB ID 5FCW). The ligand is represented as stick model (green = carbon, blue = nitrogen, red = oxygen, grey = polar hydrogens).

Since we found that the optimized HDAC11-AlphaFold model in complex with TSA and the lowest energy rotamer of Phe152 (flipped-out conformation) showed the best results in docking of selective ligands such as FT895 and SIS17, we used this model for virtual screening in the current study. To better understand the structural basis of the HDAC11 inhibition, we analyzed the shape of the binding pockets of the crystal structures and the HDAC11 AlphaFold model. The analysis revealed that the flipping of Phe152 in HDAC11 together with the less bulky residue Phe141 as foot pocket gatekeeper allows for a wider binding pocket that can accommodate the bulky methoxy substituent in the ortho position of the benzohydroxamate moiety of the hit **9**. Analysis of the crystal structures of HDAC1 (5ICN) and HDAC6 (5EDU) (Figure 6A,B) shows that, here, the different conformation of this conserved phenyl alanine brings it closer to the residues from loop 1 and loop 2 (such as Tyr24 and Lys31 in HDAC1 and Glu502 in HDAC6) and narrows the pocket in HDAC1 as well as HDAC6. As a result, this pocket cannot accommodate ortho-substituted benzohydroxamates (no zinc chelation is possible) like the hit compound **9**.

The HDAC8 crystal structure 5FCW was used as an “anti-target” for virtual screening in this study, as, to our knowledge, it has the best resolution for a wild-type human HDAC8 crystal structure co-crystallized with a hydroxamic acid. A closer look and comparison of the docked poses of the hit compound in HDAC11 and HDAC8 show that the ligand in the HDAC11 pocket is oriented slightly differently (Figure 6C,D), allowing for a better fit to the ortho substitution. Another observation is that in the docking poses in HDAC8, a considerable portion of the ligand is exposed to the solvent due to the shorter loop 1 of HDAC8, whereas the ligand in HDAC11 is stabilized by the longer loop 1, as shown in the MD studies. In the case of HDAC8-selective inhibitors, a more L-shaped conformation was observed in docking studies and X-ray structures [45,55,56]. Consideration of these observations may explain the preferential binding of the hit compound in HDAC11.

### 2.5. Molecular Dynamics Simulations

Docking methods are limited by not considering the flexibility of the protein but treating the receptor as rigid body. On the other hand, the MD simulation technique takes into account the flexibility of the complex, thus providing a deeper insight regarding the binding mode of the ligand and its behavior in a dynamic environment. Therefore, we decided to study the binding mode of the confirmed hit extensively, using short and long MD simulations. The docking pose of the hit compound in the optimized HDAC11 AlphaFold model was subjected to three short (50 ns) molecular dynamics simulations using different random seeds. Furthermore, a longer MD simulation (500 ns) was performed to assess the stability of the obtained pose over a longer time scale.

**Figure 7:**
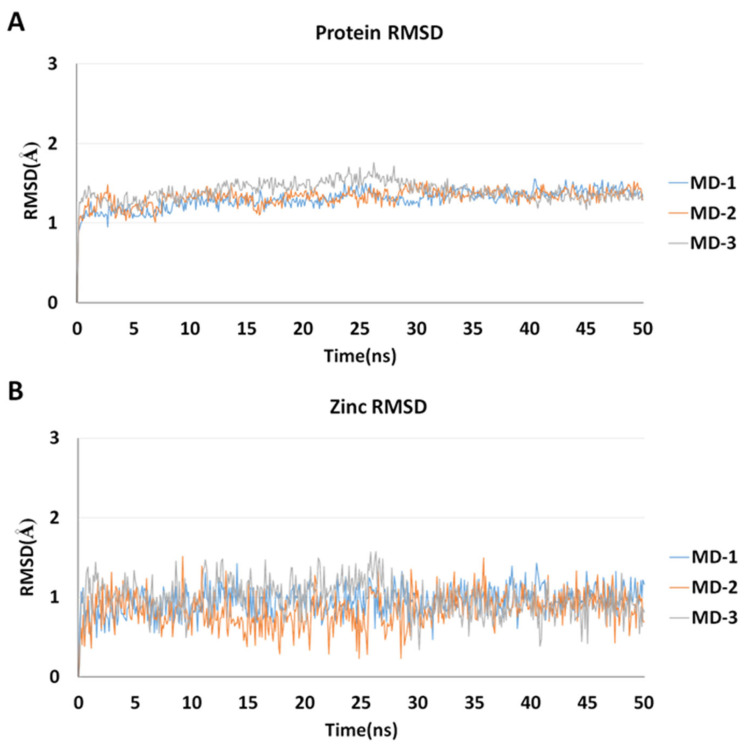
RMSD plots of ZINC000028464438 (**9**) for 3 repeated MD runs each for 50 ns. (**A**) RMSD plots of protein backbone-heavy atoms. (**B**) RMSD plots of zinc ion.

In all MD simulations, the protein and the zinc ion demonstrated high stability that could be observed through the calculated RMSD plots. The protein backbone stabilizes between 1 Å and 2 Å while the zinc ion stabilizes at almost 1 Å (Figure 7A,B).

**Figure 8:**
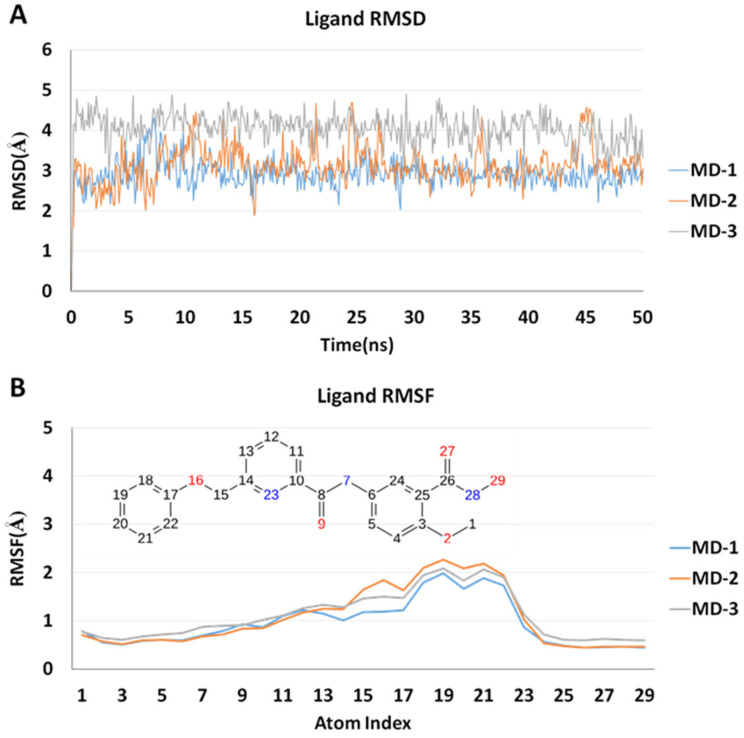
RMSD and RMSF plots of ZINC000028464438 (**9**) for 3 repeated MD runs each for 50 ns. (**A**) RMSD plots of ligand-heavy atoms. (**B**) RMSF plots of ligand-heavy atoms.

The results of the three independent short MD simulations were comparable. The RMSD plot of the ligand demonstrated that there is a shift in the pose directly after the simulation started and that the ligand stabilizes between 3 Å and 4 Å till the end of the simulation (Figure 8A). Analyzing the RMSF of the ligand-heavy atoms showed that the phenoxymethyl capping group is the most fluctuating substructure of the ligand, with an RMSF reaching 2 Å (Figure 8B).

**Figure 9:**
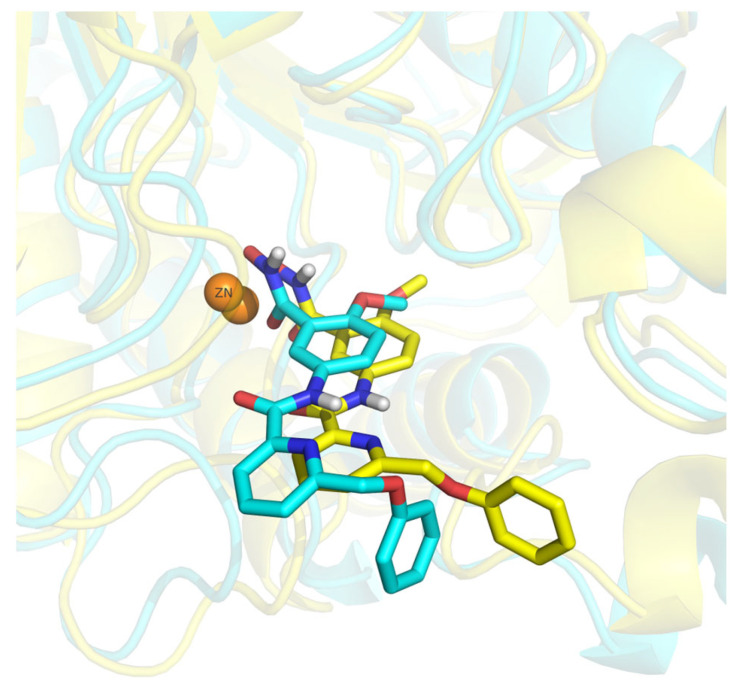
Superposition of the first and last frames of ZINC000028464438 (**9**), showing the shift in the pose during the simulation from the first MD run of 50 ns. The zinc ion is represented as orange sphere, the protein backbone as cartoon, and ligand as sticks (cyan and yellow = carbon, blue = nitrogen, red = oxygen, grey = polar hydrogens). The protein backbone and the ligand are colored in cyan and yellow for the first and last frames, respectively.

Inspecting the MD trajectories showed that there is a slight shift of the initial docking pose, allowing for the benzohydroxamate moiety to be accommodated deeper into the binding pocket, which also leads to a better accommodation of the capping group through the relaxation of the conformation (Figure 9).

**Figure 10:**
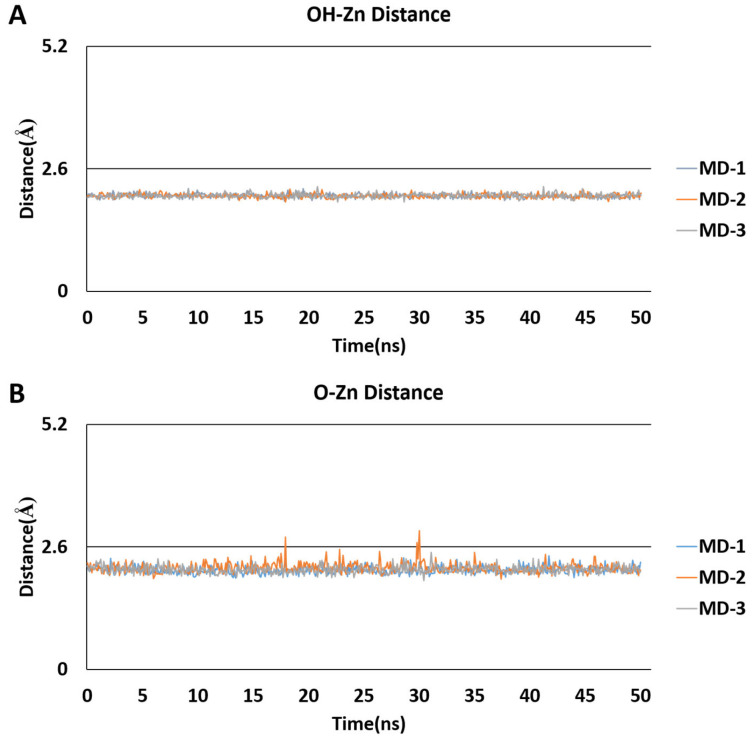
(**A**,**B**) Distances to the zinc ion for three repeated MD runs each for 50 ns for the hydroxyl and the carbonyl oxygen atoms of the hydroxamate zinc binding group, respectively.

The stability of the bidentate chelation mode was confirmed for the three runs by monitoring the distances between the chelator atoms of the hydroxamate zinc binding group and the zinc ion (Figure 10A,B). The salt bridge to His142 showed very high stability, with persistence of almost 100% for the three runs. The hydrogen bond interaction to His143 showed moderate stability, with persistence ranging between 54% and 72%. It is worth noting that we observed similar weak to moderate stability of the hydrogen bond interaction with His143 during MD simulation with some of the ligands we utilized for the model optimization in our previous study, such as TSA and some of the selective docked ligands, e.g., FT895 and MIR002 [32].

The slight shift in the pose discussed above leads to almost complete loss of the hydrogen bond between Tyr304 and the carbonyl oxygen of the hydroxamate moiety but allowed for the formation of another hydrogen bond between the same residue and the oxygen of the methoxy substituent in the ortho position of the benzohydroxamate substructure that showed high stability, with persistence ranging between 72% and 87%. This shift in the pose also allowed for the formation of another hydrogen bond interaction that was not observed in the initial docked pose between His183 and the carbonyl oxygen of the amide linker; however, low stability of this interaction was observed with persistence between 26% and 37%. (Table S2, Supplementary Materials).

**Figure 11:**
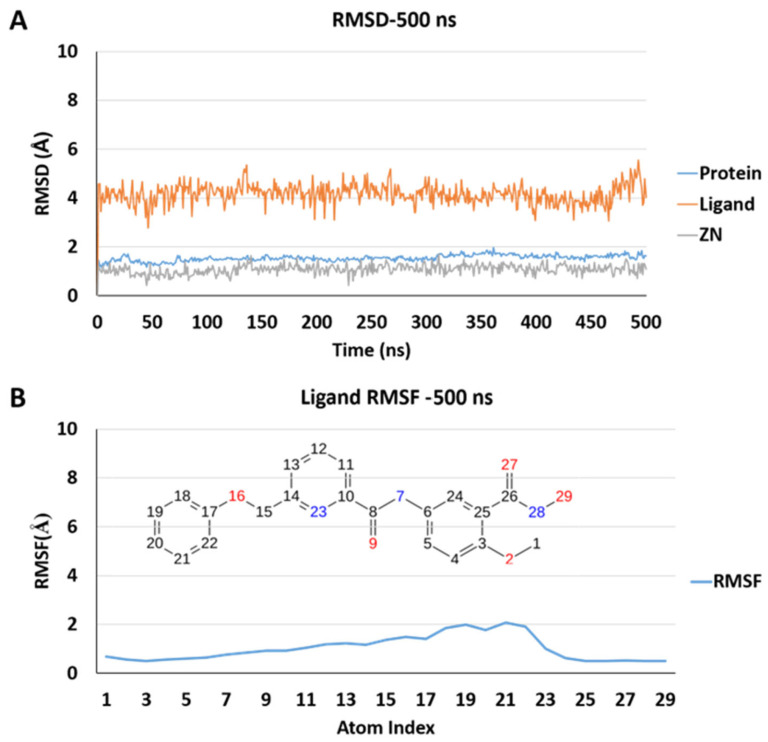
(**A**) RMSD plots of the protein backbone-heavy atoms, zinc ion, and ligand-heavy atoms for the long MD run (500 ns). (**B**) RMSF plots of the ligand- and ZINC000028464438 (**9**)-heavy atoms for the long MD run (500 ns).

The longer molecular dynamics simulation was able to confirm the stability of the obtained pose of the hit in the HDAC11 AlphaFold model over a long time scale. Inspecting the RMSD plot of the ligand showed that it is stabilizes between 4 Å and 5 Å (Figure 11A), with the RMSF indicating that the substructure that fluctuates most is the phenoxymethyl group (Figure 11B).

**Figure 12:**
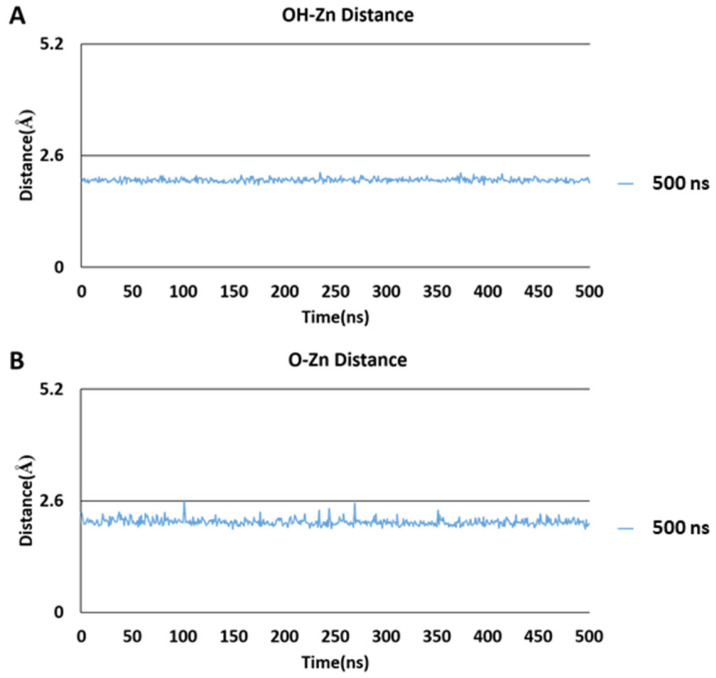
(**A**,**B**) Distances to the zinc ion for the hydroxyl and the carbonyl oxygen atoms of the hydroxamate zinc binding group, respectively, for the long MD run (500 ns).

Distances between the zinc ion and the chelator atoms of the hydroxamate zinc binding group were shown to be stable, thus confirming the bidentate chelation mode (Figure 12A,B).

**Figure 13:**
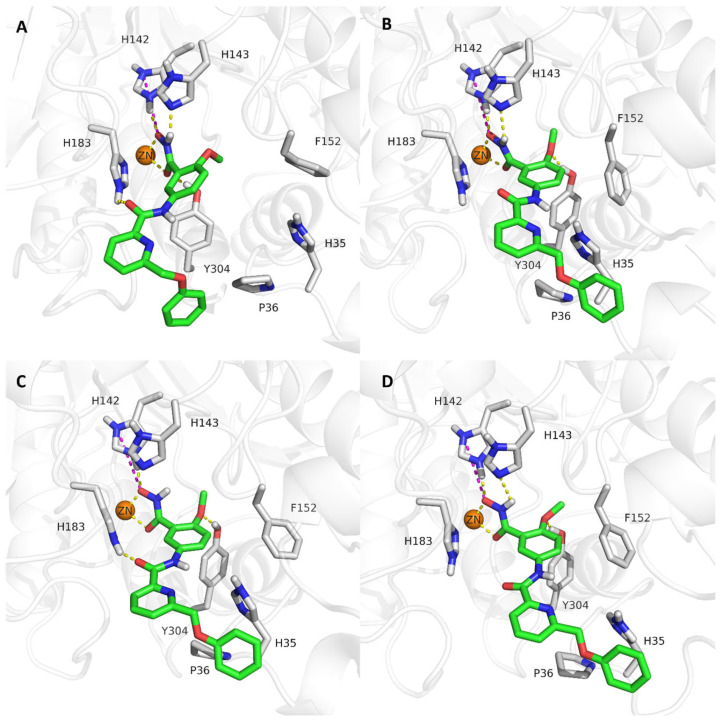
Selected snapshots from the long MD simulation (500 ns) of ZINC000028464438 (**9**)-HDAC11-docked pose showing the shift in the pose and fluctuation in the phenoxymethyl capping group. (**A**) Frame 1. (**B**) Frame 1250. (**C**) Frame 2500. (**D**) Frame 5000. The protein backbone is shown as white cartoon, zinc ion as orange sphere, the binding site residues as grey sticks, and the ligands as atom-colored sticks (green = carbon, blue = nitrogen, red = oxygen, grey = polar hydrogens). Coordination and hydrogen bonds are shown as yellow dashed lines and the ionic interactions as magenta dashed lines.

MD simulation trajectory analysis demonstrated the same slight shift in the pose, with the benzohydroxamate moiety inserted deeper into the binding pocket, along with the relaxation of the phenoxymethyl capping group (Figure 13), as observed in the three independent and shorter MD runs.

**Figure 14:**
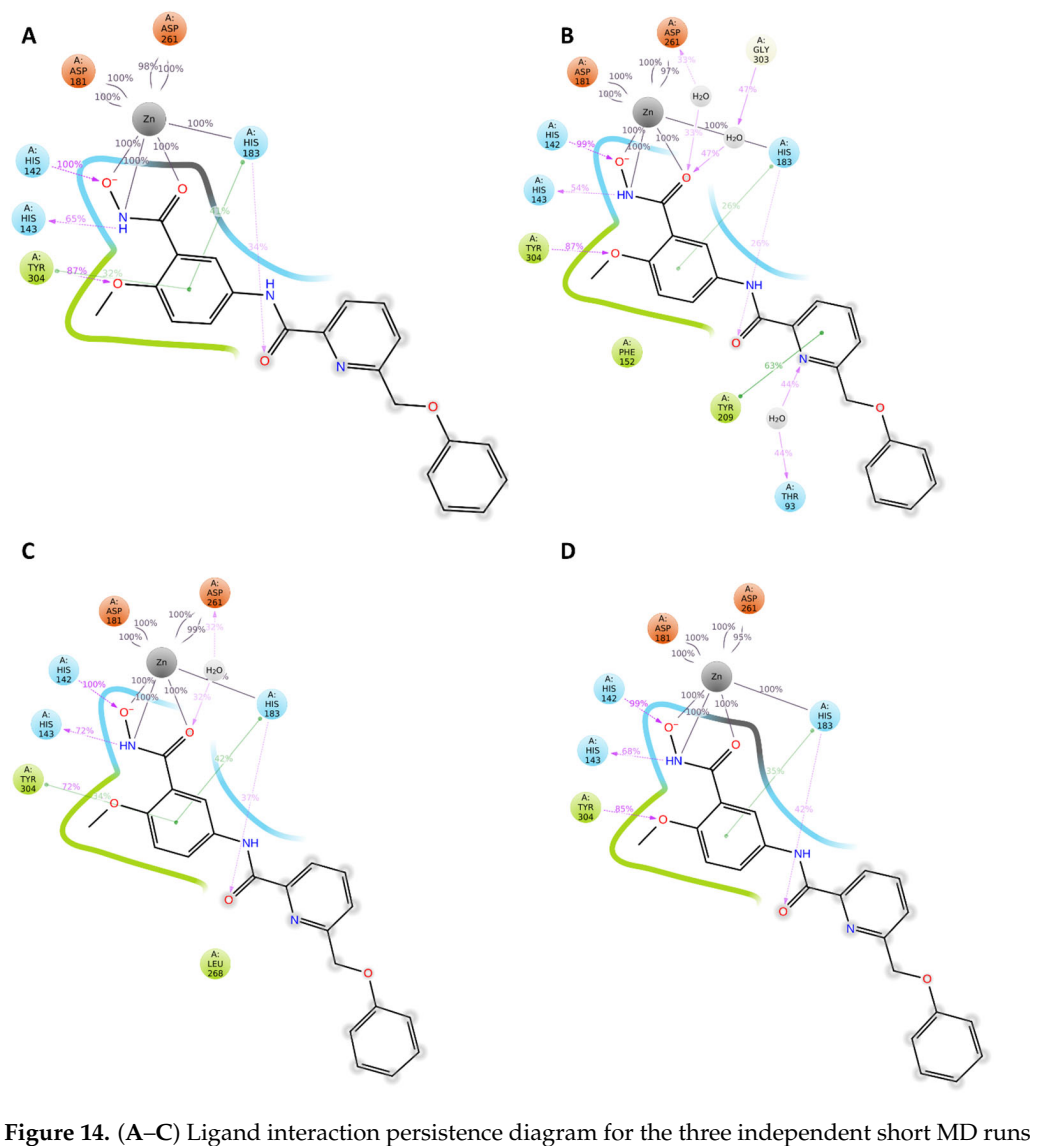
(**A**–**C**) Ligand interaction persistence diagram for the three independent short MD runs (**A**) MD1, (**B**) MD2, (**C**) MD3) (50 ns) and (**D**) the long MD run (500 ns), respectively, of HDAC11-ZINC000028464438 (**9**).

**Figure 15:**
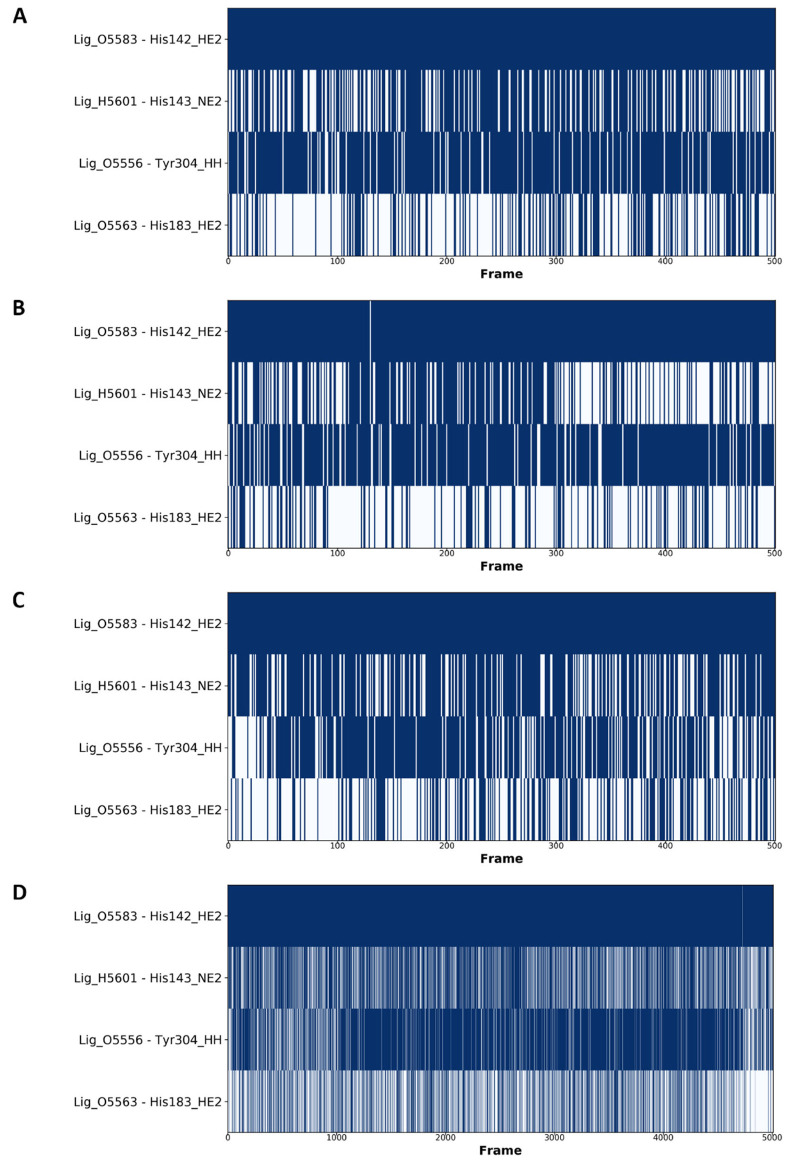
(**A**–**C**) Hydrogen bond occupancy diagrams for the three independent short MD runs (50 ns, (**A**) MD1, (**B**) MD2, (**C**) MD3) and (**D**) the long MD run (500 ns), respectively, of HDAC11-ZINC000028464438 (**9**).

The salt bridge between the deprotonated hydroxyl oxygen of the zinc binding group and His142 showed very high stability, with persistence of about 100%, while, for His143, the hydrogen bond interaction with the carbonyl oxygen of the hydroxamate moiety demonstrated average stability, with persistence of 68%. The same observations could be made concerning the other hydrogen bond interactions during the simulation in the short runs. The hydrogen bond interaction between the oxygen of the methoxy group in the ortho position to the hydroxamate moiety and Tyr304 demonstrated persistence of 85%, while for His183, a weakly stable hydrogen bond with the carbonyl of the amide linker showing persistence of 42% could be observed. Overall, the predicted binding mode of the hit compound demonstrated good stability during the MD simulation. The key interactions of the zinc binding group were not affected by the slight shift of the ligand from the initial docked pose or the fluctuation in the capping group (Figure 14 and Figure 15).

Metadynamics is an enhanced sampling technique that is able to capture the structural dynamics more efficiently in limited time scale by using a history-dependent bias potential as a function of a collective variable [57]. This process helps the system escape energy minima and previously sampled regions, thus accelerating sampling of the entire complex free-energy landscape.

Binding pose metadynamics (BPMD) application [58] implemented in Schrödinger was originally developed to rank docking poses of a single ligand in a single protein binding site by running a series of metadynamics simulations. We utilized this methodology to further explore the stability of the predicted binding mode observed for the hit compound in the HDAC11 AlphaFold model.

For this purpose, and because we observed a slight shift in the original docked pose during the classical MD simulation, we applied BPMD for the obtained docked pose and the last frame (500 ns) of the classical MD simulation representing the equilibrated ligand pose.

The BPMD method employs the RMSD of the ligand from its initial pose as a collective variable. The stability of the protein ligand complex is evaluated in terms of the ligand’s RMSD fluctuations and the persistence of important contacts between the ligand and the receptor over the course of the simulation. The PoseScore indicates the average RMSD of the ligand, the persistence score (PersScore) indicates the persistence of the interactions over the course of the simulation, and the composite score (CompScore) combines the PoseScore and PersScore [58,59].

**Figure 16:**
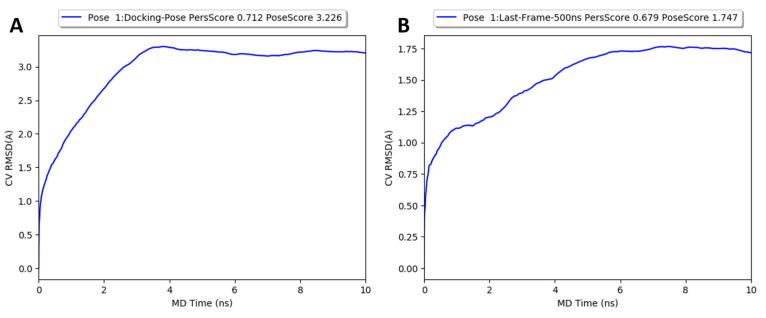
Plots of the average value of the collective variable (RMSD) over the metadynamics simulation. (**A**) The original docked pose and (**B**) the pose from last frame of the 500 ns MD simulation, respectively, of HDAC11-ZINC000028464438 (**9**).

The results from BPMD demonstrated a PoseScore of 3.226 and 1.747 for the original docked pose and the last MD frame, respectively (Figure 16). Generally, ligand poses with a PoseScore ≤ 2 Å were considered stable [58]. The resulting PoseScore indicates that the stabilized pose during the MD simulation is more stable when compared to the starting docked pose, thus reinforcing the results obtained from the classical MD simulation which showed a slight shift of the ligand during the run.

The resulting persistence of the interactions is almost equivalent for both poses and showed a PersScore of 0.712 and 0.679 for the original docked pose and the last MD frame pose, respectively. The results match the defined threshold of ≥0.6 [58], indicating that the contact network was maintained during the course of the simulation. The CompScore for the original pose and the last frame pose of the hit ligand were found to be −0.335 and −1.647, respectively, with increasingly negative values indicating better stability.

Overall, the results from the metadynamics studies confirmed the stability of the predicted binding pose in terms of the ligand RMSD and persistence of the observed interactions and further supported the results from the classical MD simulations.

## 3. Materials and Methods

Schrödinger Suite 2019 was used for all of the modeling work. Maestro [60] was utilized for visualization (Release 2019-1, Schrödinger, LLC: New York, NY, USA).

All ligands were docked in the deprotonated hydroxamate form while the grids for docking were all generated with the His142 (HDAC11 numbering) in the protonated HIP form. According to our experience from our previous study [32], this methodology shows better performance with the docking software used, Glide (Release 2019-1, Schrödinger, LLC: New York, NY, USA), in terms of reproducing the bidentate chelation native poses of the co-crystallized ligands.

### 3.1. Protein Preparation

All protein structures were preprocessed using Protein Preparation Wizard (Release 2019-1, Schrödinger, LLC: New York, NY, USA) [61,62] by adding hydrogen atoms and assigning bond orders. Water molecules beyond 5 Å from the ligands were deleted and zero order bonds to metals were added. Filling in missing side chains and loops using Prime [63,64,65] was performed. Ionization states of the ligands were generated using Epik (Release 2019-1, Schrödinger, LLC: New York, NY, USA) [66,67,68] at pH 7.0 ± 2.0. The deprotonated hydroxamates form [32,69,70,71,72] was selected for further hydrogen bond optimization. Hydrogen bond optimization was assigned with sampling water orientation, using PROPKA (Release 2019-1, Schrödinger, LLC: New York, NY, USA) at pH 7.0.

### 3.2. Grid Generation

For all protein–ligand complexes, grids were generated using the Receptor Grid Generation panel, utilizing the centroid of the ligand as the center of the grid.

### 3.3. Ligand Preparation

Ligands were prepared in the predominant form at pH 7, utilizing the LigPrep [73] panel with OPLS3e force fields.

### 3.4. Database Acquiring and Curation

#### 3.4.1. Acquiring Ligand Database

A focused library of benzohydroxamic acids (SMARTS=C1=CC=C(C(=O)NO)C=C1) comprising 407,834 ligands was downloaded from https://tldr.docking.org/ using the zinc20-all database (accessed on 1 August 2023) [49].

#### 3.4.2. Ligand Preparation

The library was prepared using Ligprep and resulted in 510,529 structures using OPLS2005 (Release 2019-1, Schrödinger, LLC: New York, NY, USA) [74,75,76,77]; possible states were generated at pH 7.2 ± 2 using Epik. Specified chiralities from the original dataset were retained.

#### 3.4.3. Property Calculation

The rule of five properties was calculated for all ligands in the database using QikProp (Release 2019-1, Schrödinger, LLC: New York, NY, USA) [78] properties from the Molecular Descriptor panel.

#### 3.4.4. Database Filtering

The prepared library was filtered to select the hydroxamate form [32,69,70,71,72] of the ligands using a defined custom pattern of [O-]N([H])C(=O)c1ccccc1. The library was filtered using the calculated rule of five properties, thereby discarding all structures which showed one or more violations of the rule of five, using the Ligand Filtering panel. A total of 18,113 compounds successfully passed the aforementioned filters.

### 3.5. Virtual Screening

#### 3.5.1. Structure-Based Pharmacophore Modeling

##### Pharmacophore Generation

The E-pharmacophore [79,80] hypothesis was generated using the Develop Pharmacophore Model panel form in Phase (Release 2019-1, Schrödinger, LLC: New York, NY, USA) [81,82,83] utilizing the optimized AlphaFold TSA-HDAC11 complex with the flipped-out Phe152 rotamer [32]. The auto E-pharmacophore method was used to specify the maximum number of features to be generated and assign the receptor-based excluded-volume shell.

##### Pharmacophore Screening

The prepared database was screened through Phase Ligand Screening panel using the previously generated E-pharmacophore and implementing the four obtained features and excluded volumes. Up to 50 conformers were generated during the search and specifying, leading us to report, at most, one hit per ligand. A total of 12,154 hits successfully passed the pharmacophore screening.

#### 3.5.2. Docking into HDAC11 AlphaFold Model

The hits obtained from the pharmacophore screening were docked into the HDAC11 AlphaFold model using Glide (Release 2019-1, Schrödinger, LLC: New York, NY, USA) [84,85,86,87] with standard precision and flexible ligand sampling. A total of 15 poses were subjected to post-docking minimization and reporting of the top-scored pose. A total of 12,151 compounds could be successfully docked.

#### 3.5.3. Pose Filtering

The obtained docking poses in the HDAC11 AlphaFold model were filtered using Pose Filter panel, utilizing the distance between the carbonyl and the hydroxyl oxygens of the hydroxamate moiety and the zinc ion while specifying maximum contact distance to be 2.6 Å. A total of 11,409 poses successfully passed the filter.

#### 3.5.4. Docking and Pose Filtering in Other HDACs’ Isoforms

The following crystal structures were used for the docking studies in other HDAC subtypes:
**Isoform****PDB ID****Resolution****Organism****Bound Inhibitor**HDAC15ICN3.30 ÅHomo sapiensHydroxamic acid inhibitorHDAC65EDU2.79 ÅHomo sapiens CD2Hydroxamic acid inhibitorHDAC85FCW1.98 ÅHomo sapiensHydroxamic acid inhibitor

##### Validation by Redocking of the Native Ligand

To validate the docking protocol, redocking of the co-crystallized ligands of HDAC1, HDAC6, and HDAC8 was performed, and RMSD for the docked and the native poses was calculated. RMSD was found to be 2.018 Å, 1.192 Å, and 0.416 Å for HDAC1, HDAC6, and HDAC8, respectively.

##### Docking and Pose Filtering

The filtered poses from the HDAC11 docking results were further docked into HDAC1, HDAC6, and HDAC8. The obtained docking poses were further subjected to Pose Filter. Ligand docking and pose filtering were performed using the same settings as mentioned for HDAC11. In total, 450, 9934, and 11,308 hits were successfully docked to HDAC1, HDAC6, and HDAC8, respectively. Compounds that were able to show correct poses and zinc chelation in HDAC1, HDAC6, and HDAC8 were removed from the final HDAC11 inhibitor hit list.

### 3.6. REOS Filtering and MM-GBSA Calculations

To remove compounds with reactive groups that may interfere with biological evaluation, rapid elimination of swill (REOS) filter was applied using structure filter in Canvas [88,89,90].

To prioritize the hits for further evaluation, ligand binding energies were calculated using the molecular mechanics with Generalized Born and surface area solvation (MM-GBSA). For this purpose, the Prime MM-GBSA panel was utilized, the variable-dielectric Generalized Born (VSGB) solvation model was specified, and sampling was carried out by minimizing all atoms using OPLS3e force field.

### 3.7. Molecular Dynamics Simulation

The predicted binding mode of the virtual screening hit of HDAC11 was further analyzed by means of molecular dynamics simulation using the program Desmond [91,92]. The HDAC11-inhibitor complex was simulated for 50 ns, and the simulation was repeated three times, applying different random seeds. Furthermore, a single longtime scale MD run was performed for 500 ns. The system was solvated in SPC water model using an orthorhombic box and a buffer distance of 10 Å distance between the solute structures and the simulation box boundary. The box volume was then minimized. The system was neutralized by adding chloride ions that were placed 4 Å away from the ligand.

Relaxation of the prepared system was performed using the default Desmond relaxation protocol for NPT ensemble followed by a production run utilizing the NPT ensemble at 300 K using a Nosé–Hoover chain thermostat and a pressure of 1.01325 bar using Martyna–Tobias–Klein barostat.

The Simulation Event Analysis panel was utilized for the calculation of RMSD and distance to the zinc ion. The RMSD of the protein was calculated using the backbone atoms, while the RMSD of the ligand and the zinc ion was calculated by fitting to the protein backbone. The Simulation Interaction Diagram panel was used for analyzing the RMSF and the interaction persistence of the ligands. RMSD of the protein was calculated excluding the termini (residues: 1–14 and 321–347).

Metadynamics (implemented in the Schrödinger software, (Release 2019-1, Schrödinger, LLC: New York, NY, USA) was used to assess the stability of the original docked pose compared to the stabilized pose resulting from the 500 ns MD run. For this purpose, Binding Pose Metadynamics panel was utilized, with the default settings of 10 trials per pose each of 10 ns. Binding pose metadynamics (BPMD) application [53] implemented in Schrödinger was originally developed to rank docking poses of a single ligand in a single protein binding site by running a series of metadynamics simulations. We utilized this methodology to further explore the stability of the predicted binding modes.

### 3.8. Chemistry

#### 3.8.1. General

Materials and reagents were purchased from Sigma-Aldrich Co., Ltd. (St. Louis, MO, USA) and abcr GmbH (Karlsruhe, Germany). Solvents used during the synthesis and purification were analytically pure and dry. Thin-layer chromatography was carried out using aluminum sheets coated with silica gel 60 F254 (Merck, Darmstadt, Germany). For medium-pressure chromatography (MPLC), columns containing silica gel Biotage^®^ (Biotage, Uppsala, Sweden) SNAP ultra-HP-sphere 25 µm were used.

The purity of the hit compound was determined using high-pressure liquid chromatography (HPLC) (Figure S3) and was measured by UV absorbance at 254 nm. The HPLC system consisted of two LC-10AD pumps, a SPD-M10A VP PDA detector, and a SIL-HT autosampler from the manufacturer Shimadzu (Kyoto, Japan). For the stationary phase, Merck LiChrospher 100 RP18, 125 mm × 4 mm, 5 µm column was used. The mobile phase was composed of methanol, H_2_O, and 0.05% trifluroacetic acid.

Mass spectrometry (MS) analyses were carried out on a Finnigan MAT710C (Thermo Separation Products, Planegg/Martinsried, Germany) for the ESI MS spectra (Figure S4). High-resolution mass spectrometry (HRMS-ESI) analyses were performed with an LTQ (linear ion trap) Orbitrap XL hybrid mass spectrometer (Thermo FisherScientific, Planegg/Martinsried, Germany) (Figure S5). Varian Inova 400 (Varian, Darmstadt, Deutschland) was used to measure ^1^HNMR and ^13^CNMR spectra using deuterated dimethyl sulfoxide (DMSO-d6) as solvent. Chemical shifts were referenced to the residual solvent signals (Figures S1 and S2).

**Scheme 1:**
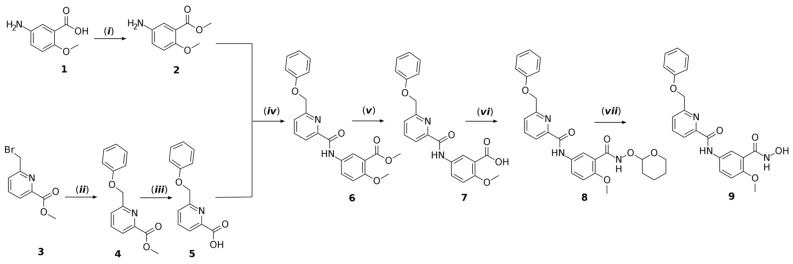
Synthesis of target compound. Reagents and conditions: (*i*) SOCl_2/_methanol/reflux/3 h; (*ii*) C_6_H_6_OH/Cs_2_CO_3_/DMF/RT/18 h; (*iii*) LiOH.H_2_O/THF:H_2_O (50:50)/RT/1 h; (*iv*) **5**/C_2_O_2_Cl_2_/DCM/RT/2 h then **2**/DIPEA/RT/overnight; (*v*) LiOH.H_2_O/THF:H_2_O (50:50)/RT/4 h; (*vi*) O-(Tetrahydro-2H-pyran-2-yl)-hydroxylamin/HATU/DIPEA/DMF/RT/4 h; (*vii*) THF/aq. HCl/RT/overnight.

The hit compound was synthesized according to Scheme 1.

#### 3.8.2. Synthesis Procedure

***Methyl 5-amino-2-methoxybenzoate hydrochloride*** (**2**).

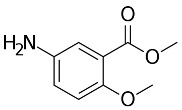



To a stirred solution of 5-amino-2-methoxybenzoic acid **1** (0.5 g, 3 mmol) in methanol, thionyl chloride, (0.33 mL, 4.5 mmol) was added dropwise. The mixture was heated under reflux for 3 h and then cooled and evaporated using rotary evaporator to afford the product as hydrochloride salt; ^1^H NMR (400 MHz, DMSO-d_6_) δ 10.29 (s, 3H), 7.64 (d, *J* = 2.8 Hz, 1H), 7.53 (dd, *J* = 8.9, 2.8 Hz, 1H), 7.24 (d, *J* = 9.0 Hz, 1H), 3.81 (s, 3H), 3.78 (s, 3H). MS *m*/*z*: [M + H]^+^ 182; yield, 98.31%.

***Methyl 6-(phenoxymethyl)pyridine-2-carboxylate*** (**4**).

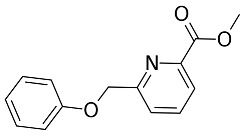



A mixture of methyl 6-(bromomethyl) picolinate **3** (1.38 g, 6 mmol), phenol (0.71 g, 7.5 mmol), and cesium carbonate (2.94 g, 9 mmol) in 20 mL DMF was stirred at room temperature for 18 h. The reaction mixture was then added dropwise to iced water and the formed precipitate was filtered and washed with water; ^1^H NMR (400 MHz, DMSO-d_6_) δ 8.06–7.95 (m, 2H), 7.77–7.70 (m, 1H), 7.33–7.23 (m, 2H), 7.06–6.97 (m, 2H), 6.97–6.88 (m, 1H), 5.22 (s, 2H), 3.87 (s, 3H). MS *m*/*z*: [M + H]^+^ 244; yield, 82.92%.

***6-(phenoxymethyl)pyridine-2-carboxylic acid*** (**5**).

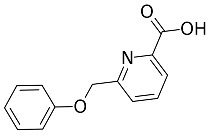



A mixture of **4** (1.21 g, 5 mmol) and lithium hydroxide monohydrate (1.05 g, 25 mmol) was stirred in a mixture of water and tetrahydrofuran (50:50) for one hour at room temperature. The reaction mixture then was added dropwise to iced water and neutralized by adding acetic acid. The mixture was then extracted with ethyl acetate and the organic layer was dried over anhydrous sodium sulfate and evaporated using rotary evaporator to afford the solid product; ^1^H NMR (400 MHz, DMSO-d_6_) δ 13.20 (s, 1H), 8.03–7.93 (m, 2H), 7.71 (dd, *J* = 7.1, 1.8 Hz, 1H), 7.33–7.24 (m, 2H), 7.07–6.98 (m, 2H), 6.97–6.90 (m, 1H), 5.21 (s, 2H). MS *m*/*z*: [M + H]^+^ 230; yield, 92.09%.

***Methyl 2-methoxy-5-[6-(phenoxymethyl)pyridine-2-amido]benzoate*** (**6**).

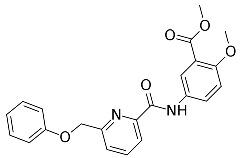



To a stirred solution of **5** (0.55 g, 2.4 mmol) in DCM, oxalyl chloride (0.26 mL, 3 mmol) was added dropwise, and the mixture was stirred for 3 h at room temperature. The mixture was then added dropwise to a solution of (0.52 g, 2.4 mmol) of **2** and N,N-diisopropylethylamine (DIPEA) (1.09 g, 8.4 mmol) in DCM, and the mixture was stirred overnight at room temperature. The reaction mixture was washed with saturated aqueous solutions of ammonium chloride and sodium carbonate followed by brine. The organic layer was then dried over anhydrous sodium sulfate and evaporated using rotary evaporator. The product was purified with medium-pressure liquid chromatography (MPLC) using mixture of n-heptane and ethyl acetate; ^1^H NMR (400 MHz, DMSO-d_6_) δ 10.51 (s, 1H), 8.19 (d, *J* = 2.7 Hz, 1H), 8.09–8.02 (m, 2H), 8.00 (dd, *J* = 9.0, 2.8 Hz, 1H), 7.73 (dd, *J* = 6.4, 2.4 Hz, 1H), 7.34–7.25 (m, 2H), 7.16 (d, *J* = 9.1 Hz, 1H), 7.09–7.00 (m, 2H), 6.98–6.91 (m, 1H), 5.32 (s, 2H), 3.80 (s, 3H), 3.79 (s, 3H). MS *m*/*z*: [M + H]^+^ 393.1; yield, 74.35%.

***2-methoxy-5-[6-(phenoxymethyl)pyridine-2-amido]benzoic acid*** (**7**).

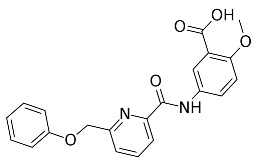



An amount (0.68 g, 1.7 mmol) of **6** was dissolved in a mixture of tetrahydrofuran and water (50:50), an amount (0.355 g, 8.5 mmol) of lithium hydroxide monohydrate was added, and the reaction mixture was stirred for 4 h at room temperature. The reaction mixture was added dropwise to iced water and neutralized by acetic acid. The solution was then saturated with sodium chloride, and the solid precipitate was filtered and washed with water; ^1^H NMR (400 MHz, DMSO-d_6_) δ 12.70 (s, 1H), 10.48 (s, 1H), 8.15 (d, *J* = 2.7 Hz, 1H), 8.10–8.01 (m, 2H), 7.96 (dd, *J* = 9.0, 2.8 Hz, 1H), 7.73 (dd, *J* = 6.8, 2.1 Hz, 1H), 7.34–7.26 (m, 2H), 7.12 (d, *J* = 9.0 Hz, 1H), 7.08–7.00 (m, 2H), 6.98–6.90 (m, 1H), 5.32 (s, 2H), 3.79 (s, 3H). MS *m*/*z*: [M + H]^+^ 379,1; yield, 94.56%

***N-{4-methoxy-3-[(oxan-2-yloxy)carbamoyl]phenyl}-6-(phenoxymethyl)pyridine-2-carboxamide*** (**8**).

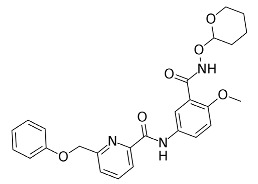



A mixture of **7** (0.57 g, 1.5 mmol) and hexafluorophosphate azabenzotriazole tetramethyl uronium (HATU) (0.68 g, 1.8 mmol) in DMF was stirred for 15 min, after which, O-(tetrahydro-2H-pyran-2-yl)-hydroxylamin (0.2 g, 1.7 mmol) and DIPEA (0.58 g, 4.5 mmol) were added and stirring was continued for 4 h. The reaction mixture was diluted with water and extracted with ethyl acetate. The organic layer was washed with saturated solutions of ammonium chloride and sodium carbonate followed by brine. The organic layer was dried over anhydrous sodium sulfate and evaporated using rotary evaporator. The product was purified using medium-pressure liquid chromatography (MPLC) using a mixture on n-heptane and ethyl acetate; ^1^H NMR (400 MHz, DMSO-d_6_) δ 11.02 (s, 1H), 10.49 (s, 1H), 8.13–8.00 (m, 3H), 7.96 (dd, *J* = 8.9, 2.8 Hz, 1H), 7.73 (dd, *J* = 6.5, 2.3 Hz, 1H), 7.35–7.25 (m, 2H), 7.12 (d, *J* = 9.0 Hz, 1H), 7.08–7.00 (m, 2H), 6.99–6.91 (m, 1H), 5.32 (s, 2H), 5.06–4.96 (m, 1H), 4.08–3.97 (m, 1H), 3.82 (s, 3H), 3.55–3.44 (m, 1H), 1.81–1.62 (m, 3H), 1.60–1.44 (m, 3H). MS *m*/*z*: [M + H]^+^ 478.2; yield, 84.8%

***N-[3-(hydroxycarbamoyl)-4-methoxyphenyl]-6-(phenoxymethyl)pyridine-2-carboxamide*** (**9**).

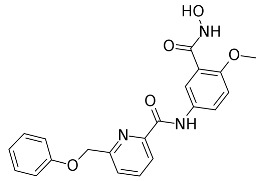



An amount (0.58 g, 1.2 mmol) of **8** was dissolved in 20 mL of tetrahydrofuran, 1 mL of 2N aqueous HCl was added, and the mixture was stirred overnight. The reaction mixture was then added dropwise to iced water, and the precipitate was filtered and washed with water; ^1^H NMR (400 MHz, DMSO-d_6_) δ 10.62 (s, 1H), 10.47 (s, 1H), 9.09 (s, 1H), 8.13–8.00 (m, 3H), 7.93 (dd, *J* = 9.0, 2.8 Hz, 1H), 7.73 (dd, *J* = 6.6, 2.2 Hz, 1H), 7.36–7.25 (m, 2H), 7.11 (d, *J* = 9.0 Hz, 1H), 7.08–7.00 (m, 2H), 6.99–6.90 (m, 1H), 5.32 (s, 2H), 3.82 (s, 3H). ^13^C NMR (101 MHz, DMSO-d_6_) δ 163.15, 162.49, 158.44, 156.55, 153.46, 149.75, 139.36, 131.68, 130.07, 124.98, 124.01, 122.77, 122.53, 121.65, 121.52, 115.20, 112.48, 70.13, 56.35. MS *m*/*z*: [M + H]^+^ 394.3, HRMS *m*/*z*: [M + H]^+^ 394.1394; calculated C_21_H_20_O_5_N_3_: 394.1403. HPLC: rt 13.123 min (purity 95.755%); yield 77.43%.

### 3.9. In Vitro Enzymatic Inhibition Evaluation

In the case of HDAC11, the full-length human was expressed and purified as described in previous work [22]. A fluorescence-based HDAC11 assay was used. The fluorescence measurements were performed using a PerkinElmer Envision 2104 multilabel plate reader (Waltham, MA, USA) at λex = 320 nm and λem = 430 nm. The reaction mixture consisted of HDAC11 and the fatty acid-acylated peptide substrate derived from TNFα in a reaction buffer—comprising 50 mM HEPES, 2 mg/mL BSA, and 70 µM TCEP—at pH 7.4, which was adjusted with NaOH (total volume 40 μL). The reactions were incubated in black 384-well plates for 30 min (scan every 30 s) at room temperature, and the increase in relative fluorescence reflecting the product formation was monitored. Positive (HDAC11, substrate, DMSO, and buffer) and negative controls (substrate, DMSO, and vuffer) were included in every measurement. They were set as 100 and 0%, respectively, and the measured values were normalized accordingly.

For HDAC1, 2, 3, 6, and HDAC6, the recombinant proteins were purchased from ENZO Life Sciences AG (Lausen, Switzerland), whereas HDAC4–7, 9, and 10 were produced as described in previous work [93]. All inhibitors were tested in an enzymatic in vitro assay (Table S3), as described previously, using 384-well plates (GreinerONe, catalogue no. 784900) [55,93]. After five minutes of incubation of inhibitors with the respective enzyme (HDAC1: 10 nM, HDAC2 and 3: 3 nM, HDAC4: 5 nM, HDAC5: 10 nM, HDAC6: 1 nM, HDAC7: 5 nM, HDAC8: 2 nM, HDAC 9: 20 nM, HDAC10: 5 nM), the reactions were started by the addition of the substrate.

For HDAC1, 2, 3, and 6, an acetylated peptide substrate derived from p53 (Ac-RHKK(Acetyl)-AMC) was used in a discontinuous fluorescence assay, as described previously [55]. All reactions were performed in assay buffer (20 mM HEPES, 140 mM NaCl, 10 mM MgCl_2_, 1 mM TCEP, and 0.2 mg/mL BSA, pH 7.4 adjusted with NaOH) at 37 °C. After 1 h, the reaction was quenched by adding trypsin and SAHA. The fluorescence intensity was measured after 1 h of incubation using an Envision 2104 Multilabel Plate Reader (PerkinElmer, Waltham, MA, USA) with an excitation wavelength of 380 ± 8 nm and an emission wavelength of 430 ± 8 nm.

HDAC4–7, 8, 9, and 10 were measured in a continuous manner using the thioacetylated peptide substrate (Abz-SRGGK(thio-TFA)FFRR-NH2) which was described previously [93]. For HDAC 10, an internal quenched spermidine-like substrate was used. The fluorescence increase was followed for 1 h with two reads per min with an excitation wavelength of 320 ± 8 nm and an emission wavelength of 430 ± 8 nm. For all measurements, positive (enzyme, substrate, DMSO, and buffer) and negative (substrate, DMSO, and buffer) controls were included in every measurement and were set as 100 and 0%, respectively. The measured values were normalized accordingly.

## 4. Conclusions

In the current study, a structure-based pharmacophore model utilizing our previously optimized HDAC11 AlphaFold model was generated as the preliminary step for screening a large, focused library of benzohydroxamate compounds. The resulting hits were further docked in an HDAC11 model and followed by pose filtration to select compounds that could show bidentate chelation of the catalytic zinc ion. A comparative approach was then applied by docking the hits obtained from docking in HDAC11 using different selected HDAC isoform (HDAC1, HDAC6, and HDAC8) crystal structures and eliminating compounds that showed good poses in other HDAC isoforms. This approach proved effective in filtering the initially obtained hit compounds to find a selective ligand. The obtained hits that showed good poses in HDAC11 but not in the other isoforms were subjected to a final filtration step using the REOS filter, and the final hits were further prioritized by MM-GBSA calculations. It is interesting to see that all top-ranked hits have a substituent in ortho-position to the aromatic hydroxamate group. This ortho-substituent is sterically accepted in the HDAC11 binding pocket only. In all other HDAC structures studied in the current work, this substitution leads to the abolition of the correct chelation of the zinc ion. The experimentally confirmed selectivity for HDAC11 underpins the usefulness of the optimized HDAC11 AlphaFold model for structure-based drug design.

Moreover, the binding mode of the confirmed hit in HDAC11 was further analyzed by several MD simulations. MD simulation studies proved the stability of the initially observed binding mode in terms of ligand RMSD, RMSF, bidentate chelation of the zinc ion, and interaction stability.

In conclusion, a multistep and comparative virtual screening approach was successfully implemented in an attempt to identify novel selective HDAC11 inhibitors utilizing a previously optimized HDAC11 AlphaFold model. This study experimentally verifies the HDAC11 AlphaFold model optimization approach we adopted in our previous study. Additionally, it confirms that AlphaFold models can be utilized for the aim of drug design and discovery subsequent to a prior optimization.

## Figures and Tables

**Table 1:** Final hits and MM-GBSA dG binding values (black = carbon, blue = nitrogen, red = oxygen, chlor = green).

Title	Structure	MM-GBSA dG Bind
ZINC000028464438 (**9**)	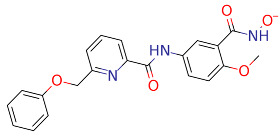	−49.76
ZINC000671998736	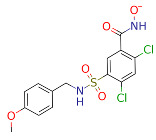	−39.75
ZINC000742823399	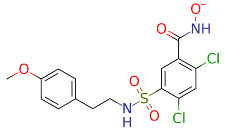	−48.68
ZINC000916666211	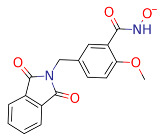	−48.21
ZINC000916666264	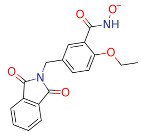	−41.45

## Data Availability

Data are contained within the article and Supplementary Materials.

## Supplementary Materials

The following supporting information can be downloaded at: https://www.mdpi.com/article/10.3390/ijms25021358/s1.
