# Supplementary material for: Comparative Structure-Based Virtual Screening Utilizing Optimized AlphaFold Model Identifies Selective HDAC11 Inhibitor
Source: Int J Mol Sci. 2024 Jan 22;25(2):1358. doi: 10.3390/ijms25021358 (PMC10816272; doi:10.3390/ijms25021358)
Supplement: Supplementary file 1 [file ijms-25-01358-s001.zip › ijms-2793050-supplementary.pdf]

Supplementary Materials

Comparative Structure-Based Virtual Screening Utilizing Optimized AlphaFold Model Identifies Selective HDAC11 Inhibitor

Fady Baselious [1], Sebastian Hilscher [1], Dina Robaa [1], Cyril Barinka [2], Mike Schutkowski [3] and Wolfgang Sippl [1,*]

[1] Department of Medicinal Chemistry, Institute of Pharmacy, Martin-Luther-University of Halle-Wittenberg, 06120 Halle (Saale), Germany; f_noshy@yahoo.com (F.B.); sebastian.hilscher@pharmazie.uni-halle.de (S.H.); dina.robaa@pharmazie.uni-halle.de (D.R.)
[2] Institute of Biotechnology of the Czech Academy of Sciences, BIOCEV, 252 50 Vestec, Czech Republic; cyril.barinka@ibt.cas.cz
[3] Charles Tanford Protein Center, Department of Enzymology, Institute of Biochemistry and Biotechnology, Martin-Luther-University of Halle-Wittenberg, 06120 Halle (Saale), Germany; mike.schutkowski@biochemtech.uni-halle.de
* Correspondence: wolfgang.sippl@pharmazie.uni-halle.de

Table of Content

Table of contents

	Topic	Page
**1**	**Structures of virtual screening hits**	**S2**
**2**	**Molecular dynamics results**	**S3**
**3**	**Analytical data**	**S4-S8**
**4**	**In vitro data**	**S9-S10**

Table S1. Virtual screening hits (7) obtained after excluding the bidentate poses in HDAC1, HDAC6 and HDAC8 as well as monodentate poses in HDAC6.

	Title	Structure
1	ZINC000028464438	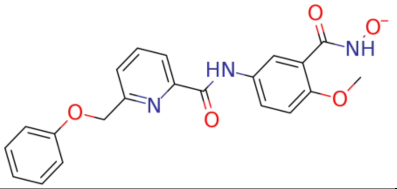
2	ZINC000671998736	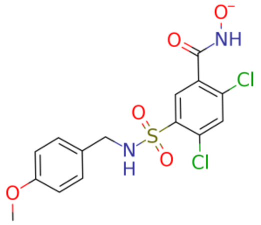
3	ZINC000725337504	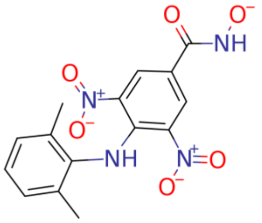
4	ZINC000742823399	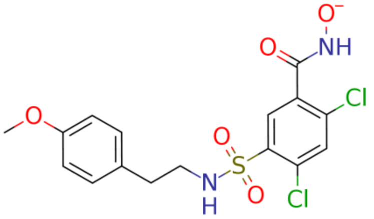
5	ZINC000787867170	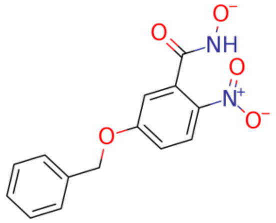
6	ZINC000916666211	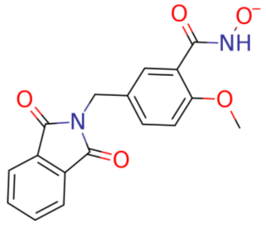
7	ZINC000916666264	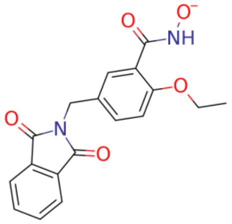

Table S2. Hydrogen bond occupancy (%) for three repeated 50 ns MD runs and one 500 ns MD run

	**His142**	**His143**	**His183**	**Tyr304**
**Run-1**	100.0	65.7	34.5	87.0
**Run-2**	99.8	54.7	26.9	87.4
**Run-3**	100.0	72.7	37.3	72.3
**500ns**	100.0	68.1	42.6	85.0

Analytical data

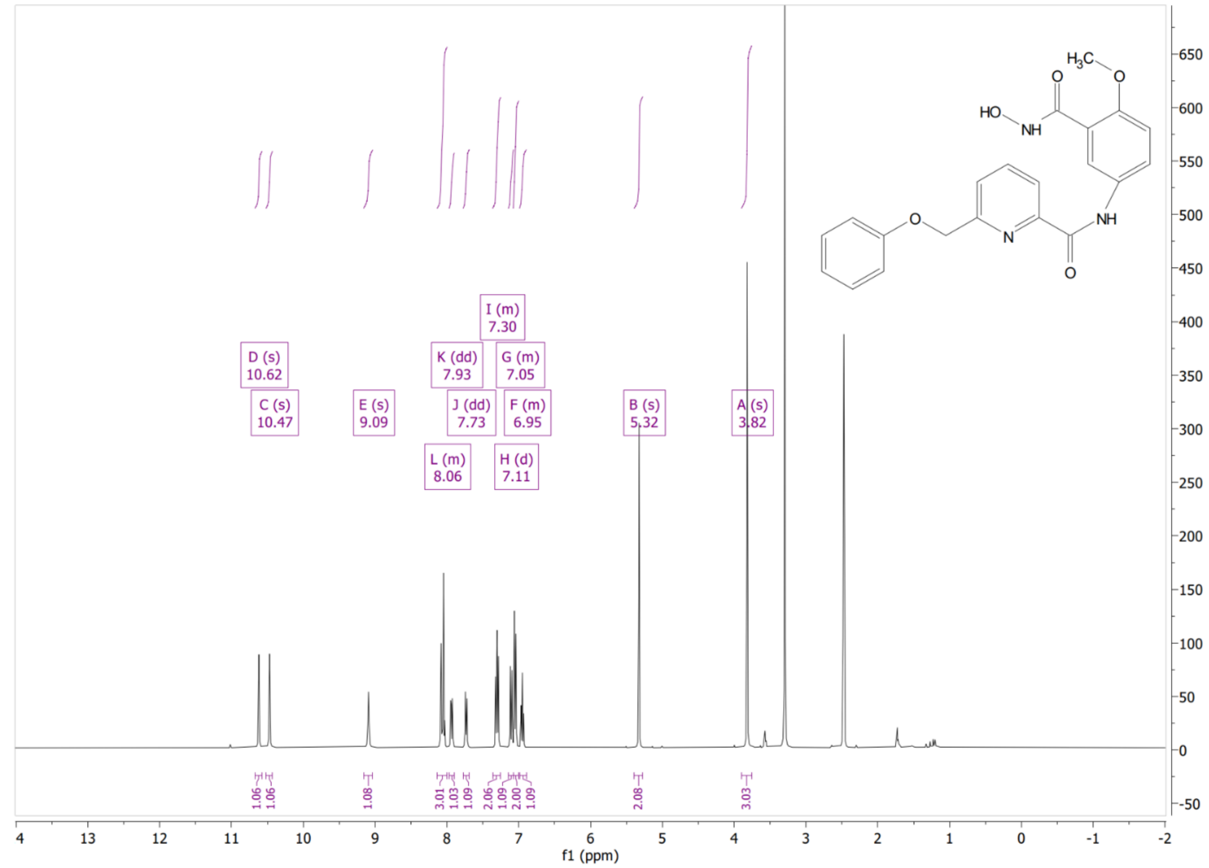

Figure S1. ^{1}H NMR chart of **1** (ZINC000028464438).

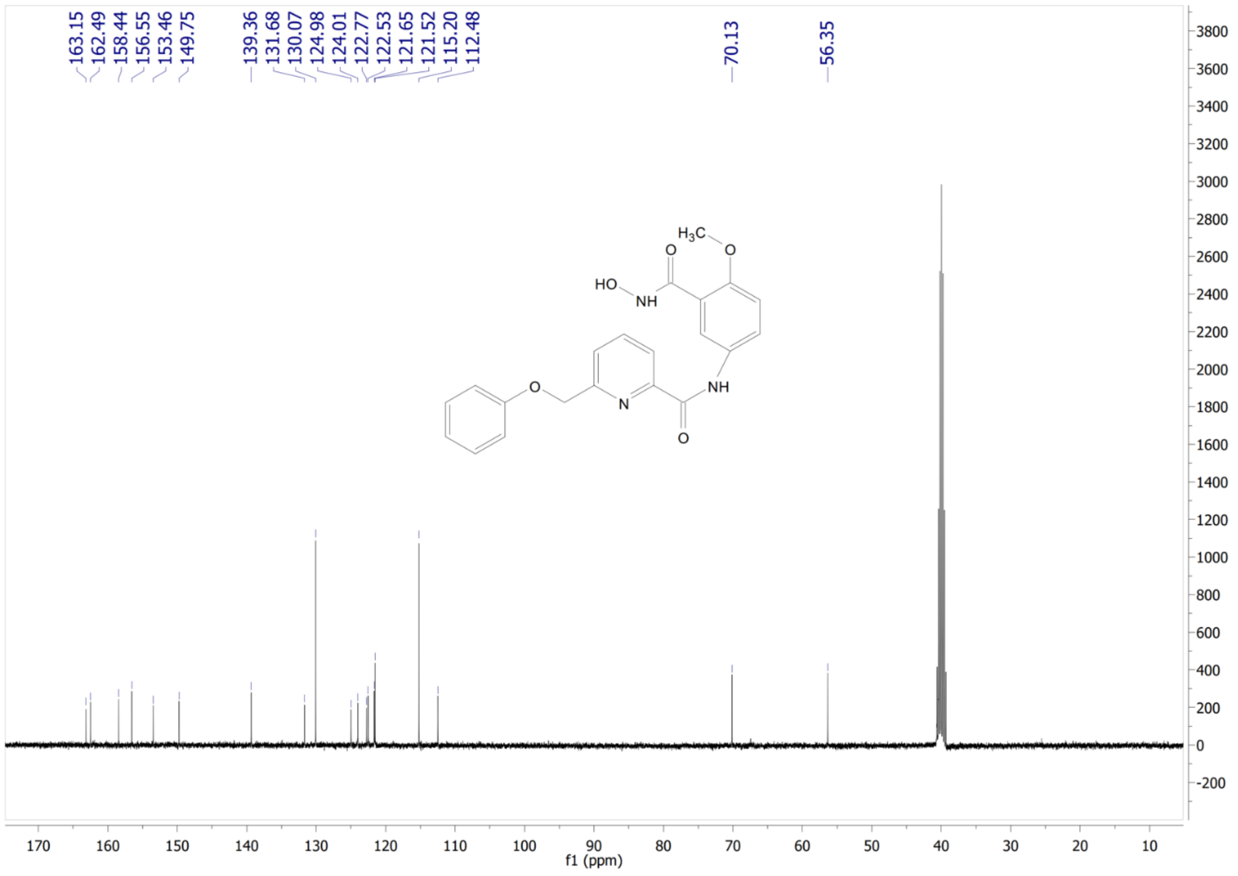

Figure S2. ¹³C NMR chart of **1** (ZINC000028464438).

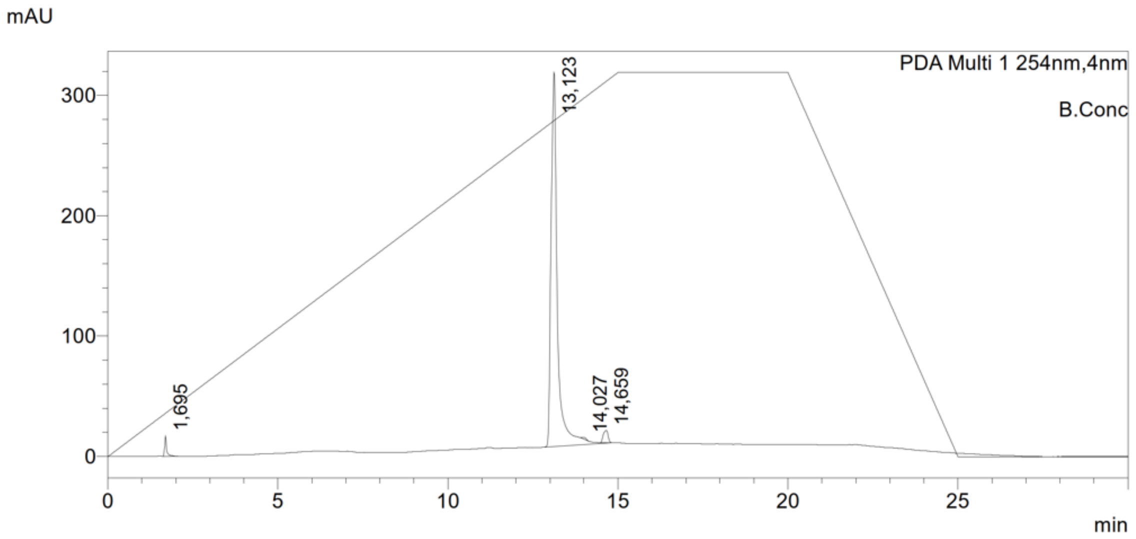

PDA Ch1 254nm

Peak#	Ret. Time	Area	Area%	Tailing Factor	Resolution(USP)
1	1,695	70031	1,668	1,812	--
2	13,123	4021050	95,755	1,540	56,157
3	14,027	9999	0,238	0,882	3,439
4	14,659	98247	2,340	0,868	2,665
Total		4199327	100,000		

Figure S3. HPLC chromatogram of **1** (ZINC000028464438).

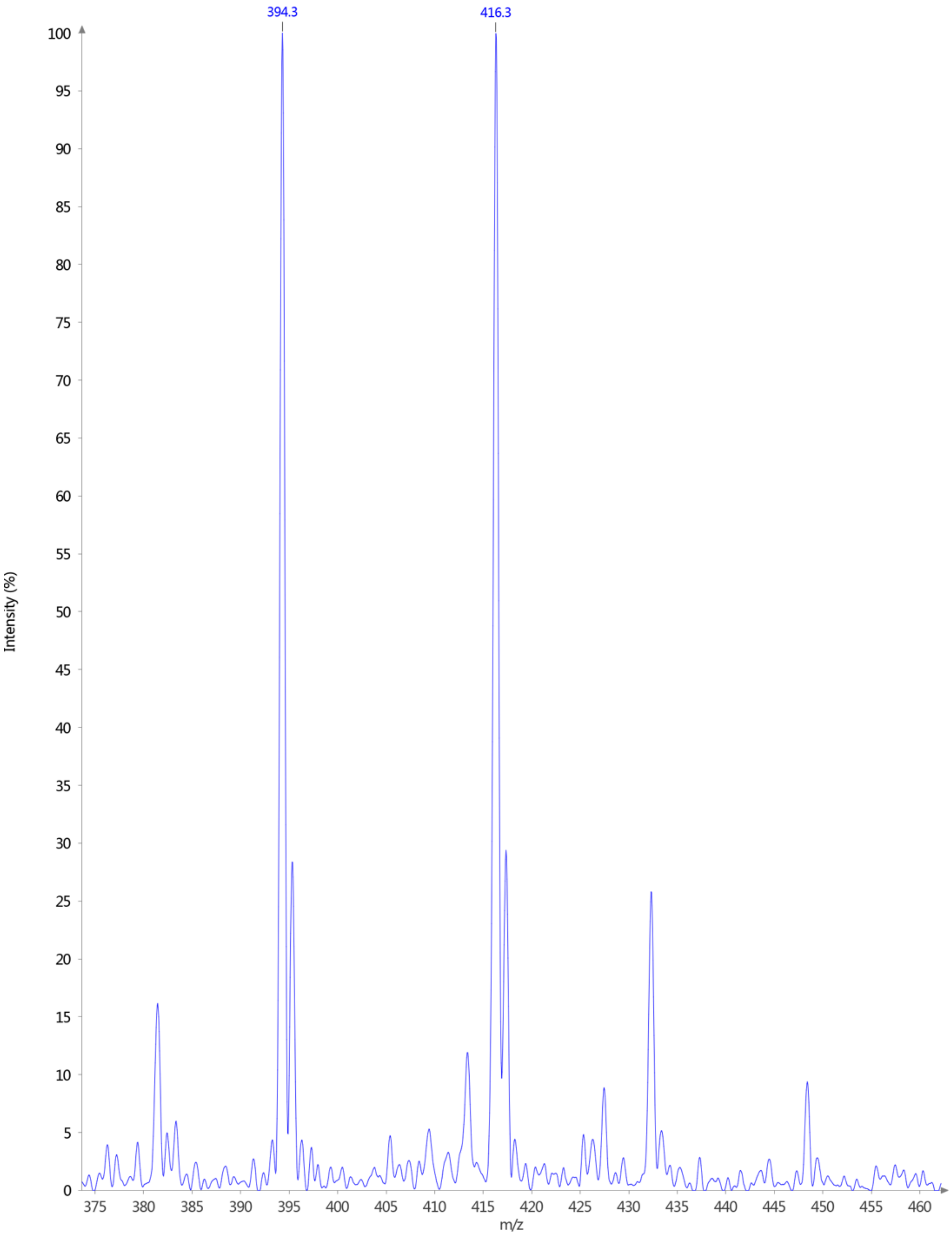

Figure S4. Mass spectrum of **1** (ZINC000028464438).

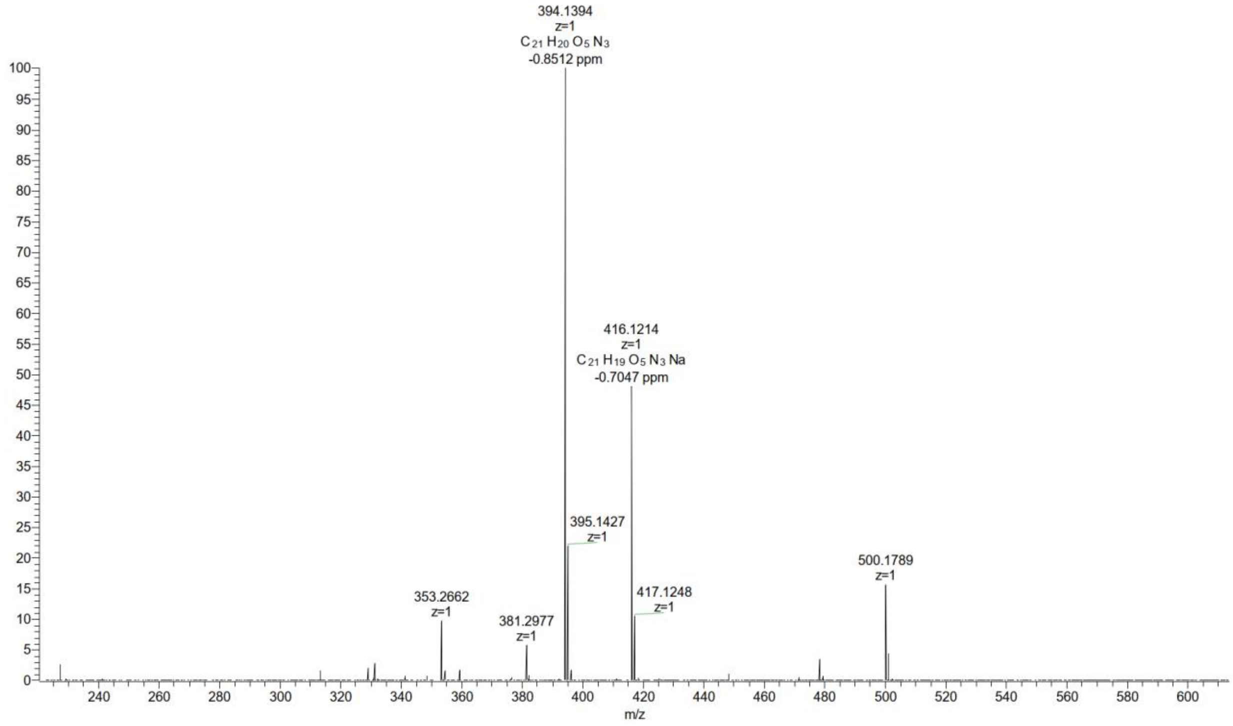

Figure S5. High resolution mass spectrum of **1** (ZINC000028464438).

In vitro data

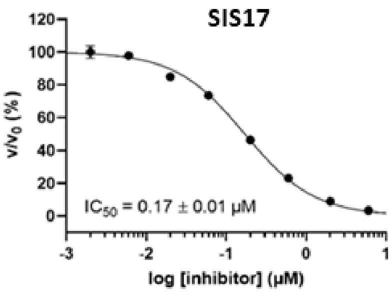

Figure S6. IC_{50} plot of the reference inhibitor SIS17 for HDAC11.

Table S3. Reference inhibitors used for the HDAC in vitro assay

IC_{50} ◎M	HDAC1	HDAC2	HDAC3	HDAC6	HDAC8	HDAC10	HDAC11
SAHA	0.101± 0.007	0.43 ± 0.009	0.21 ± 0.01	0.100 ± 0.01	0.55 ± 0.1	n.d.	n.d.
Entinostat	0.93 ± 0.1	0.95 ± 0.03	1.8 ± 0.1	>20	>20	>20	>20
Nexturastat	0.50 ± 0.030	n.d.	n.d.	0.0035 ± 0.001	0.41 ± 0.1	>10	3.25 ± 0.2
PCI-34051	28.3 ± 2.0	>20	>20	48.2 ± 6.2	0.092 ± 0.015	n.d.	n.d.
Tubastatin A	1.91 ± 0.42	n.d.	n.d.	0.034 ± 0.001	1.44 ± 0.12	0.22 ± 0.02	n.d.
SIS17	11.2% @ 10 ◎M	7.8% @ 10 ◎M	0% @ 10 ◎M	6.7% @ 10 ◎M	12.6% @ 10 ◎M	17.5% @ 10 ◎M	0.170 ± 0.02
